# Potential of Arabica Coffee Beans from Northern Thailand: Exploring Antidiabetic Metabolites through Liquid Chromatography with Tandem Mass Spectrometry (LC-MS/MS) Metabolomic Profiling across Diverse Postharvest Processing Techniques

**DOI:** 10.3390/foods12213893

**Published:** 2023-10-24

**Authors:** Cholpisut Tantapakul, Sucheewin Krobthong, Prasara Jakkaew, Wattanapong Sittisaree, Chanat Aonbangkhen, Yodying Yingchutrakul

**Affiliations:** 1 The Research Unit of Natural Product Utilization, School of Science, Walailak University, Nakhon Si Thammarat 80160, Thailand; tcholpisut@gmail.com; 2 Center of Excellence in Natural Products Chemistry (CENP), Department of Chemistry, Faculty of Science, Chulalongkorn University, Bangkok 10330, Thailand; sucheewin.k@chula.ac.th (S.K.); chanat.a@chula.ac.th (C.A.); 3 School of Information Technology, Mae Fah Luang University, Thasud, Muang, Chiang Rai 57100, Thailand; 4 Merck Life Science Thailand, Bangkok 10110, Thailand; wattanapong.sittisaree@merckgroup.com; 5 National Center for Genetic Engineering and Biotechnology, National Science and Technology Development Agency (NSTDA), Pathum Thani 12120, Thailand

**Keywords:** amylase, DPP4, chlorogenic acid, geological indicator, TLC, mass spectrometry

## Abstract

Coffee, a widely consumed beverage worldwide, undergoes postharvest methods that influence its physicochemical characteristics, while roasting modulates its composition, affecting sensory attributes. This study investigates the impact of distinct postharvest methods (washed and natural) on the antidiabetic activities, including α-amylase and DPP4, as well as the phytochemical profiling of geological indicator (GI) coffee beans (*Coffea arabica* L.). The results indicate notable differences in antidiabetic activity and phytochemical profiles between washed and natural processing methods. Coffee beans processed naturally exhibit significant suppression of DPP4 and α-amylase activities (*p*-value < 0.01) compared to beans processed using the washed technique. TLC profiling using the ratios of the solvent systems of ethyl acetate/dichloromethane (DCM) and acetone/DCM as separation solvents reveals dominant spots for the washed technique. LC-MS/MS-based untargeted metabolomics analysis using principle component analysis (PCA) clearly segregates samples processed by the natural and washed techniques without any overlap region. A total of 1114 phytochemicals, including amino acids and short peptides, are annotated. The natural processing of coffee beans has been shown to yield a slightly higher content of chlorogenic acid (CGA) compared to the washed processing method. Our findings highlight the distinct bioactivities and phytochemical compositions of GI coffee beans processed using different techniques. This information can guide consumers in choosing coffee processing methods that offer potential benefits in terms of alternative treatment for diabetes.

## 1. Introduction

Diabetes mellitus (DM) is a chronic health disease. It is generally characterized by hyperglycemia and abnormal carbohydrate metabolism. There are two main types of DM, including type 1 diabetes and type 2 diabetes (T2DM). T2DM primarily occurs and develops due to β-cells in pancreatic cell abnormality and insulin resistance, with these leading to hyperglycemia (high sugar in blood) [1]. High blood glucose levels have been found to play a crucial role in developing complications of T2DM [2]. Therefore, to slow down T2DM progression, diabetics need their blood sugar to be maintained at an optimistic level. From other views, high body fat in obesity and T2DM are closely related. Although high body fat is known to be an important factor determining the increase in the risk of T2DM, the exact mechanism of their relationship is not yet clear. One of the therapeutic routes for controlling blood glucose levels involves the suppression or inhibition of α-amylase, dipeptidyl-peptidase 4 (DPP-4), and α-glucosidase activities [3]. When food intake and physical exercise are not enough to control blood sugar, some people with T2DM turn to use synthetic drugs, for example, metformin, gliclazide, glimepiride, acarbose, and miglitol. However, metformin has common side effects, including feeling or being sick and diarrhea. In terms of other drugs, acarbose and miglitol exhibit excellent α-amylase and α-glucosidase inhibitory activities. However, they may result in abdominal distention, flatulence, vomiting, and diarrhea [4]. To solve these issues, more and more research evidence shows that natural foods from plants can also help control blood sugar and the fat content in cells.

Coffee is a popular beverage that is enjoyed by many people around the world. It is made by brewing the roasted seeds of the Coffea plant, which are often referred to as “coffee beans”. There are a number of potential health benefits associated with drinking coffee. For example, many findings also suggest that coffee may help to reduce the risk of several serious health conditions, including Parkinson’s disease, type 2 diabetes, and certain types of cancer [5,6,7]. In addition, coffee is also a good source of antioxidants, which can help to protect the body against the damaging effects of free radicals [8,9]. Polyphenols, quercetin, and CGA are among the most promising phytochemicals found in coffee beans, offering potential health benefits, such as combating obesity and DM [10,11]. In Thailand, the coffee industry has a long history and plays an important role in the country’s economy. The main coffee-growing regions in Thailand are located in the north of the country, with the main varieties being Arabica and Robusta [12]. Coffee beans can vary significantly in terms of their flavor, aroma, and other characteristics. The geographical origin of the beans can be an important factor in these differences [13]. Coffee is grown in many different countries around the world, and the specific conditions in each region can have a big impact on the flavor and quality of the beans. For example, coffee grown at high altitudes tends to be more flavorful and aromatic than coffee grown at lower altitudes [13]. The type of soil, the climate, and other environmental factors can also play a role in the characteristics of the coffee beans [14]. In addition, the processing method used and the roast level of the beans can impact the flavor and quality of the coffee [15,16]. As a result, it is common for coffee to be labeled by its origin, and many coffee lovers are interested in trying different processed types of coffee from different regions to compare the flavors and characteristics.

Coffee bean harvesting is the process of collecting the ripe coffee cherries from the coffee plant. There are three main methods of coffee bean harvesting: natural (dry), wash (wet), and honey (semidry) processing. First, the natural process is the cheapest, oldest, and simplest processing method. This processing uses only natural sunlight or a mechanical drier to dry whole coffee beans so that they fully absorb and maintain most of their various flavors and substances [17]. Therefore, this postharvesting technique makes different characteristics from each cultivation area. Natural processed coffee provides a fruity flavor and sweeter seed. This process takes time because the entire coffee cherry takes some time to dry compared to the drying of coffee beans in the washed process. The coffee beans obtained by dry processing have a heavy body, softness and sweetness, and complex characteristics. Second, the washed process is the most popular type of postprocessing. It is a water-based process. Water is needed in large quantities (40 L/kg of dry coffee weight) to perform the washed process [18]. This postprocessing was developed in an area where coffee beans cannot be dried in the sun like the natural process. During the process, green coffee beans show several enzymatic and metabolic activities [19]. These biochemical changes are mainly caused by the germination process, and the stress metabolism causes significant differences in the chemical composition of green coffee beans and determines the quality of the coffee beans [19]. By using water in processing, it has a clean taste with acidity and sourness in the style of coffee. Third, honey processing is an intermediate process between natural and wash processing. This processing has less body compared to naturally processed coffee beans. The quality of honey-processed coffee is bright and clean and is somewhat similar to wash-processed coffee beans. In addition to the aforementioned processes, there are many different, detailed methods in the world of coffee production due to each of the cultivation areas being slightly different in terms of geography and climate. For all the above-mentioned, coffee beans undergo various postharvest steps, and many metabolites that are related to the quality of coffee are affected and changed [16,20,21]. Different processed coffee beans have a significant impact on the quality of the resulting coffee. Both the natural and washed methods of postharvest processing that are chosen can influence the flavor, aroma, body, and acidity of coffee. The choice of method depends on the desired metabolite composition. Coffee extracts can provide a rich source of information through metabolomics studies. Different methods of coffee processing can result in different levels of biologically active compounds in the final product. Metabolomics can be used to identify these differences and evaluate the potential health effects of different coffee processing methods. This could provide insight into the factors that contribute to the flavor and other characteristics of the coffee of the different processing techniques. Our research aims to identify the specific types and quantities of chemicals found in coffee following various postharvest processing methods, including natural and washed, within a designated coffee-growing region. Additionally, we are investigating their potential antidiabetic properties. In essence, this research seeks to advance our scientific comprehension of coffee chemistry, thereby shedding light on its multifaceted implications for sensory perception and health outcomes.

## 2. Materials and Methods

### 2.1. Sample Collection and Preparation

Arabica coffee beans were harvested from specific GI (Moo.6, Pa-Rai, Tepsadej, Doisaket, Chiang-Mai, Thailand) with geological coordination (18.920486, 99.356228), which is a coffee farmer’s field. The coffee beans were harvested between 13th and 27th February 2021. Coffee processing converts freshly collected coffee cherries into highly desirable green coffee beans ready for roasting. The coffee’s taste and qualities depend on the processing method. The intricate process begins with skilled harvesters selecting fully developed cherries with a brilliant red or deep purple color, indicating their optimal ripeness. Depulping then carefully removes the cherry’s exocarp or outer peel. Mechanical depulping or hand-cranking can remove coffee beans from cherries. They reveal the beans under mucilage, a sticky saccharine substance. Washing coffee involves carefully fermenting mucilage-coated beans in water-filled tanks or bins. This mechanism aids in the breakdown and removal of the mucilage layer. After fermentation, the beans are washed thoroughly to eliminate any leftover mucilage. After washing or depulping, the beans are carefully placed in thin layers on patios, elevated beds, or drying racks to dry naturally or mechanically. This crucial stage lasts several days until the beans reach an ideal moisture content of 10–12%. A specialized machine hulls desiccated beans to remove the parchment coating during washing. This fragile, paper-like coating encases the beans. Washed and natural processed beans, which retain some mucilage, are turned and raked during drying to facilitate equal drying and prevent mold growth. Washed and natural processed beans are milled to remove their skin, pulp, and parchment after drying. Dehydrated legumes, regardless of processing method, are carefully sorted to remove any defects, like discolored or damaged beans. Finally, the sorted beans are graded by size, weight, and appearance. This rigorous assessment is followed by meticulous bagging of the beans for export or distribution. It is important to remember that these processes, along with regional elements like climate, altitude, and soil composition, create coffee’s diverse flavor profiles. Thus, every processing method gives the final cup of coffee its own qualities, making coffee processing a critical step in the coffee production process.

For consistency in this experiment, the beans were sort based on their size. (Coefficient variation <20% was cut-off criteria.) The coffee beans with different postharvest processing techniques were ground in liquid nitrogen and extracted using hot water extraction (95 °C) for 20 min, with mixing at 200 rounds per minutes. The effect of both postharvested coffee beans on antidiabetic activities, including DPP4 and α-amylase activity, was determined. The phytochemical profiling of both postharvested coffee beans was also investigated using TLC and LC-MS/MS.

### 2.2. Batch Pilot Configurations for Coffee Bean Processing and Phytochemical Extraction

After the coffee beans were harvested and prepared as detailed in Section 2.1, the coffee beans were extracted and processed in order to be used for brewing coffee. A total of 100 g of coffee beans was extracted and processed using in-house natural or washed processing methods. Briefly, for natural process, 100 g of coffee beans were left to dry in the sun on aluminum foil for 48 h, with the outer layers of the coffee beans still intact. After 48 h, the beans were dried and then hulled to remove the outer layer of the beans. For the washed process, the outer skin and mucilage of 100 g of coffee beans were roughly removed. The beans were put in the fermentation tank for removing outer skin and mucilage with a water stream for 24 h at 37 °C. For the final steps, the beans were dried under incubator for 48 h. Ten grams of both processed coffee methods were mechanically ground using a hand grinder.

To obtain the coffee extracts for experimental measurements, the ground coffee beans were then extracted in 100 mL of methanol (Sigma Aldrich Co., St. Louis, MO, USA) in 0.05% formic acid (Sigma Aldrich Co.) at −20 °C for 48 h. After that, C18 solid phase extraction (C18-SPE) (Waters Co., Milford, MA, USA) was conducted to clean up the samples. A total of 90 mL of methanolic fraction of both extracted coffee bean conditions was loaded on the equilibrated C18-SPE. The metabolites were absorbed on the C18 (solid material) and cleaned using 100 mL of deionized water. The elute solvent (95% acetonitrile in 0.1% formic acid) was loaded into C18-SPE and collected the fraction. The eluted fraction was dried using speed vacuum and subjected to enzyme-based experiments (DPP4 and α-amylase), cell-based experiments (cell cytotoxicity and lipid accumulation), and phytochemical profiling (TLC and LC-MS/MS).

### 2.3. DPP4 Activity Determination

Effect of both processed coffee extracts on DPP4 activity was continually measured using fluorescence detection following manufacturer’s protocol (Cayman DPP IV screening kit) (Cayman Chemical, MI, USA) with slight modification. Briefly, the master mix of the reaction was prepared by mixing the DPP4 assay buffer and substrate (gly-pro-7-amino-4-methyl coumarin) at ratio 19:1 (*v*/*v*). The testing samples (natural and washed processed coffee beans) were diluted with deionized waters at ratio 1:10 (*v*/*v*) before the experiment. Then, 2 μL of testing samples and 2 μL of deionized water (blank) were added to each of the sample wells in 96-well flat-bottom plate. The master mix (40 μL) was added to each of the sample wells and incubated at 37 °C for 30 min. To control the reaction, sitagliptin was used as the standard inhibitor (composed of 10 μL of DPP4 assay buffer and 10 μL of sitagliptin). To estimate the DPP4 activity, the 96-well plate measured the fluorescence intensity (λ_ex_ = 360 and λ_em_ = 460 nm) using microplate reader (Multiskan Go, Thermo Scientific, Waltham, MA, USA). The percentage of DPP4 activity was calculated using following Equation (1):

%DPP4 activity = [(λ_em_ of blank − λ_em_ of testing sample)/λ_em_ of blank] × 100% (1)

where λ_em_ of blank is emission at 460 nm without testing samples, and λ_em_ of testing samples is emission at 540 nm in treatment with testing samples. The reaction was conducted with 3 biological replications and 3 technical replications.

### 2.4. α-Amylase Inhibitory Activity Determination

The pancreatic porcine α-amylase inhibition was determined using dinitro-salicylic acid (DNS) as described. Briefly, 1 mg of dried coffee bean extract in both processing conditions was solubilized in 10 mL of assay buffer (10 mM sodium phosphate buffer with 5 mM NaCl, pH 6.9) at 50 °C for 10 min. A total of 10 μL of extract in both processing conditions was added to 50 μL of α-amylase (0.5 U/mL) (Sigma Aldrich Co.). The reactions were incubated at 37 °C for 5 min, and 140 μL of 0.5% starch solution (prepared in the assay buffer) was added and incubated at 37 °C for 30 min. To stop the enzyme activity, the reaction was incubated at 95 °C for 10 min. After that, 0.9 mL of DNS was added. The reaction was diluted with deionized water at 1:5 (*v*/*v*) ratio. The absorbance was measured using microplate reader at 540 nm. Percentage of inhibition of enzyme activity was calculated as (2):

%Amylase inhibition = [(λ_540_ of blank − λ_540_ of testing sample)/λ_540_ of blank] × 100 (2)

where λ_540_ of blank is absorbance at 540 nm without testing samples, and λ_540_ of testing samples is absorbance at 540 nm in treatment with testing samples. The known inhibitor (acarbose) at 10 μg/μL was used to control the reaction. The reaction was conducted with 3 biological replications and 3 well replications.

### 2.5. TLC Profiling of Different Processed Coffee

Phytochemical profiling was performed using precoated TLC plates (silica gel 60 F254, 20 × 20 cm, Merck Co., Darmstadt, Germany). A total of 0.1 microgram of the extracted samples was dissolved in DCM (Sigma Aldrich Co.) and applied onto the TLC plates (Sigma Aldrich Co.) using micropipette. An applied TLC plate was presaturated with the mixture of solvents with different mobile phases (Table 1) in the glass chamber at room temperature.

After the solvent ran up to ~90% of total TLC plate height, the plate was air dried and stained with p-anisaldehyde, followed by heating of the plate. The developed TLC plate was scanned under visible light using image scanner software.

### 2.6. Sample Preparation and LC-MS/MS Setting for Untargeted Metabolomics Analysis

The dried processed coffee by natural and washed methods (as detailed in Section 2.2) was reconstituted in 1000 μL methanol in 0.1% formic acid before being subjected to LC-MS/MS analysis. Metabolite profiling was analyzed using LC-MS/MS following previous protocol with minor modifications [22]. Briefly, an analytical column, Hypersil GOLD™ column (Thermo Fisher Scientific, Waltham, MA, USA), held at 50 °C, was used during the analysis. A total of 2 µg (4 µL) sample injections (concentration = 0.5 µg/µL) was used at a flow rate of 0.35 mL/min. The mobile phase was composed of 80%/20% methanol/water with 0.1% formic acid (MP: A) and acetonitrile with 0.1% formic acid (MP: B) (LC-MS grade, Sigma). Gradient starting conditions were 99% MP: A and 1% MP: B. Starting conditions were held for 1 min before rising to 55% B over 18 min. The column was flushed with 99% MP: B for 6 min before returning to the starting conditions. The total time of each analysis was 35 min. MS was operated in a positive mode. A spray voltage of 3.8 kV in both positive, sheath gas, and auxiliary gas flow rates were set at 48 and 11 arbitrary units (AU), respectively. The capillary temperature was 350 °C. The MS analysis alternated between MS full scans and data-dependent MS/MS scans with dynamic exclusion. LC-MS for full MS: scan range, 60–700 *m*/*z*; resolution 120,000; AGC target 3 × 10^6^; max. IT 50 ms and LC-MS for full MS/MS:, resolution 30,000; AGC target 1 × 10^5^; max. IT 100 ms. Up to ten ions with the most intense signal were fragmented. To prevent sample contamination, a blank sample (0.1% formic acid/water) was administered after every injection. All LC-MS runs were acquired using Xcalibur 3.1 software (Thermo Scientific).

### 2.7. Data Processing for Untargeted Metabolomics Analysis

The acquired raw MS files were processed using Compound Discoverer 3.1 (Thermo Fisher Scientific) to identify phytochemicals. Peak identification, peak alignment, and peak feature extraction were all conducted in a positive mode on the data. The retention time (RT) and *m*/*z* of different injections were conducted according to the retention time deviation of 0.5 min and the mass deviation of 5 ppm. Then, the peak extraction was performed according to the set information and adduct information: mass deviation = 5 ppm, signal strength deviation = 30%, signal-to-noise ratio = 2, and fine isotopic pattern matching >90% of the precursor and the characteristic product ions. Additionally, the peak area was quantified. The target *m*/*z* ions were then integrated to predict the molecular formula, which was compared to mzCloud (https://www.mzcloud.org; accessed on 15 September 2023) and ChemSpider (http://www.chemspider.com; accessed on 15 September 2023) online databases for the identification and confirmation of the compounds. Among candidate metabolites obtained from mzCloud and ChemSpider, the highest MS/MS coverage scores were selected for annotation. The PCA was conducted using Plotly version 2.0 (R package).

### 2.8. Absolute Quantifying of CGA Using LC-MS/MS

A stock solution of CGA (50 ppm or 0.05 mg/mL) (Sigma Aldrich Co.) was freshly prepared in methanol (LC-MS grade) using amber vials. Working solutions of these compounds were then prepared by diluting the above stock solution with 0.1% formic acid/methanol (*v*/*v*) for the purpose of analytical method validation and standard curve construction. The working solutions covered a concentration range from 1 ppm to 0.075 ppm. For the analytical method validation and standard curve construction, the stock solutions were appropriately diluted to obtain the working solutions within the specified concentration range. An aliquot of 3 µL from each working solution was injected into the LC-MS/MS analysis. Quantification was performed using LC-MS/MS. The LC conditions and settings were consistent with the configuration used for untargeted metabolomics analysis method. MS was operated in positive mode. LC-MS acquisitioned in parallel reaction monitoring (PRM) used the following settings: MS2 resolution 30,000; AGC target 2 × 10^5^; max. IT 150 ms. The precursor product ion used for PRM for each analyte was obtained at *m*/*z* = 355.10236 (C_16_H_18_O_9_) (NCE = 45) for chlorogenic acid. The samples were injected (n = 3) with above-mentioned parameter in LC-MS/MS. Standard curve of quercetin was constructed using the nominal known concentrations and was plotted against the corresponding peak areas. In order to absolutely quantify metabolites of CGA in the samples, the samples were reanalyzed in PRM acquisition mode. Each sample (natural and washed coffee beans) was quantitatively analyzed by comparing extracted ion chromatograms for each metabolite in Xcalibur 4.0 Quan Browser software (Thermo Fisher Scientific). The quantitation algorithm used suitable area of product ion spectra from the known metabolites’ standard concentration. The experiment was conducted with three biological replicates and three technical replicates, resulting in a total n = 9 samples per experimental group.

### 2.9. Statistical Analysis

All experiments were carried out with at least three independent replicates (n = 3), and all data were expressed as means ± standard deviation. The significance in differences was determined using Duncan’s multiple range test (*p*-values < 0.05). The absolute quantification of CGA is presented as the mean of duplicate extractions ± standard deviation.

## 3. Results

### 3.1. Coffee Bean Harvesting

**Figure 1:**
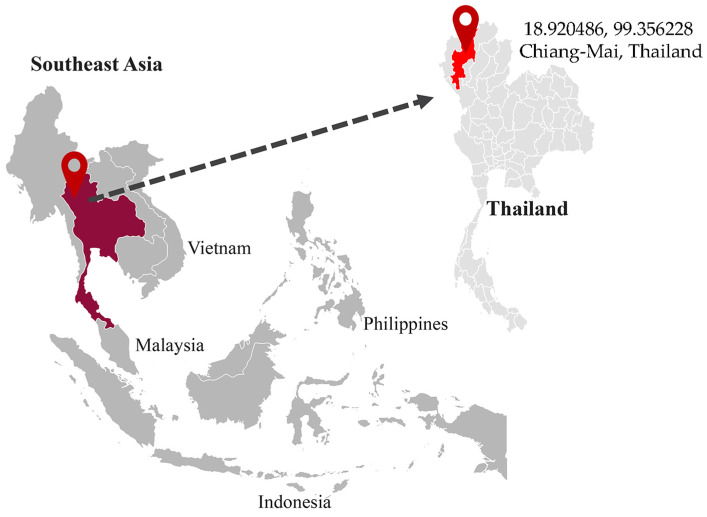
The planting location of coffee beans. The coffee beans were harvested at a specific location in Northern Thailand.

The coffee beans were sourced from a distinct geographical location, with coordinates (18.920486, 99.356228) situated at Moo. 6, Pa-Rai, Tepsadej, Doisaket, Chiang-Mai, Thailand (as depicted in Figure 1).

The one-year average temperature and humidity recorded were 14.71 °C and 67.71%, respectively. To ensure consistency in the results without the influence of varying tree ages, coffee trees aged over 30 years (<35 years) were selected.

### 3.2. DPP4 Activity Determination

**Figure 2:**
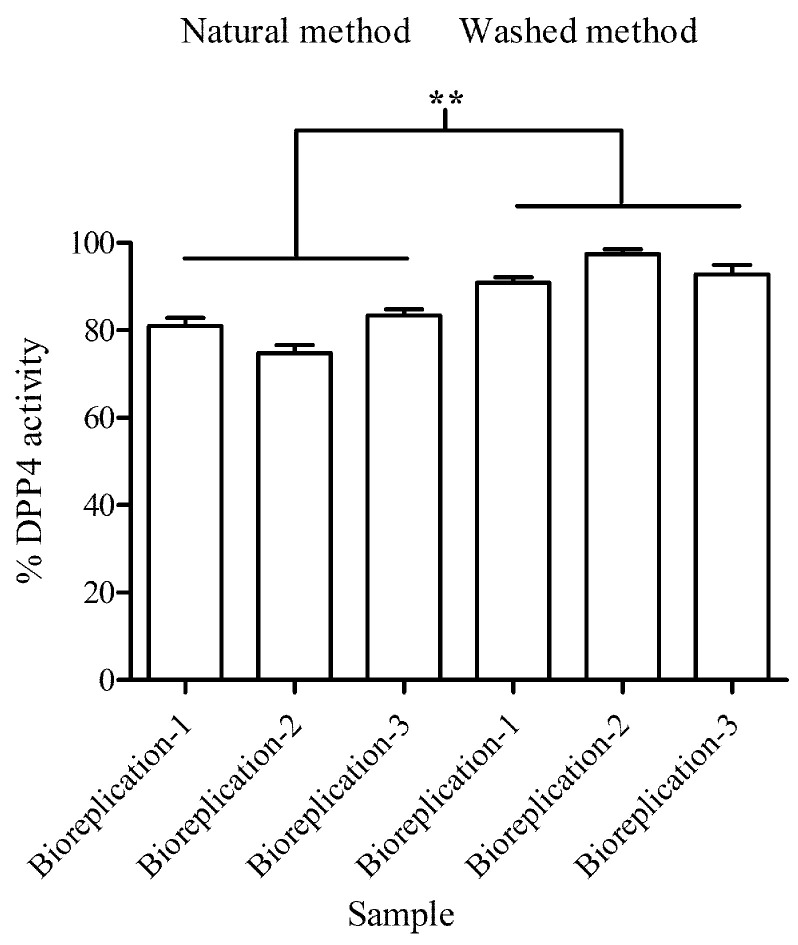
The level of DPP4 activity in processed coffee beans was measured using fluorescent-based quantification. The measuring was conducted in three independent experiments (with three biological replicates each) and included three technical replicates for each experiment. Two processing methods, namely, natural and washed methods, were utilized for the measurement. The symbols (**) indicates *p*-value < 0.01.

To investigate DPP4 activity in both processed coffee beans, we monitored the fluorescent product of DPP4 and used it to calculate the percentage of enzyme activity remaining. In the present study, the processed beans using natural and washed methods exhibited 20.12 ± 2.34% and 12.1 ± 1.94% remaining activity compared to the control group (*p*-value < 0.01), respectively (Figure 2).

In a comparison between different processed beans, the natural processed beans significantly exhibited higher DPP4 inhibition than the washed processed beans. For the control condition, the known DPP4 inhibitor (sitagliptin) significantly inhibited DPP4 activity 66.76% ± 1.04% compared to the control group (*p*-value < 0.01).

### 3.3. α-Amylase Inhibitory Activity

**Figure 3:**
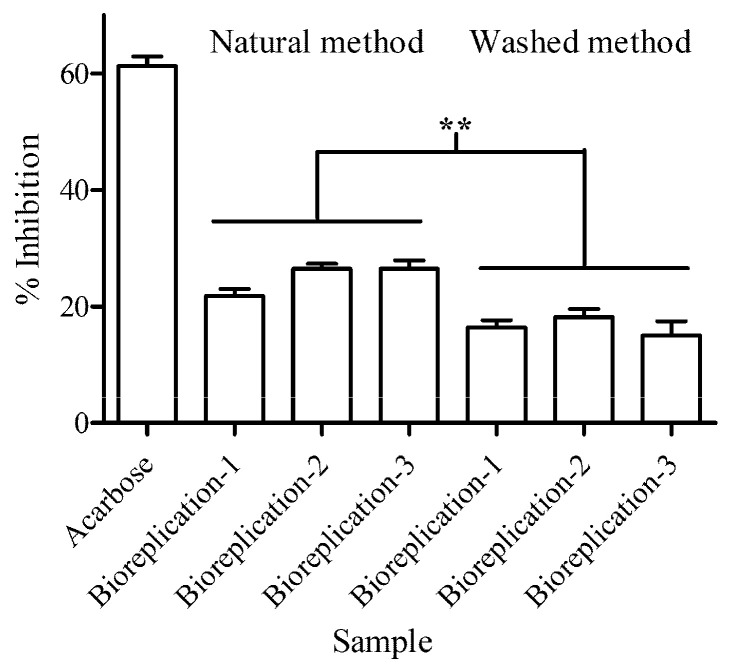
The level of α-amylase inhibitory activity in processed coffee beans was measured using colorimetric-based quantification. The measuring was conducted in three independent experiments (with three biological replicates each) and included three technical replicates for each experiment. Two processing methods, namely, natural and washed methods, were utilized for the measurement. Acarbose was used as a known α-amylase inhibitor control. The symbols (**) indicates *p*-value < 0.01.

To investigate the effect of both processed bean methods on α-amylase activity, we measured the reduction in DNS and used it to calculate the percentage of α-amylase inhibition. The processed beans using the natural method exhibited 22.12% ± 1.94% inhibition, while the washed method exhibited 19.23% ± 1.94% inhibition compared to the control group (Figure 3). In this study, acarbose was taken as a positive control; it exhibited the highest inhibition level at 60.12 ± 1.87%.

For comparative inhibition activity between different processed beans, the natural processed beans significantly exhibited higher DPP4 inhibition than the washed processed beans (*p*-value < 0.01).

### 3.4. Metabolite Profiling Analysis Using TLC

**Figure 4:**
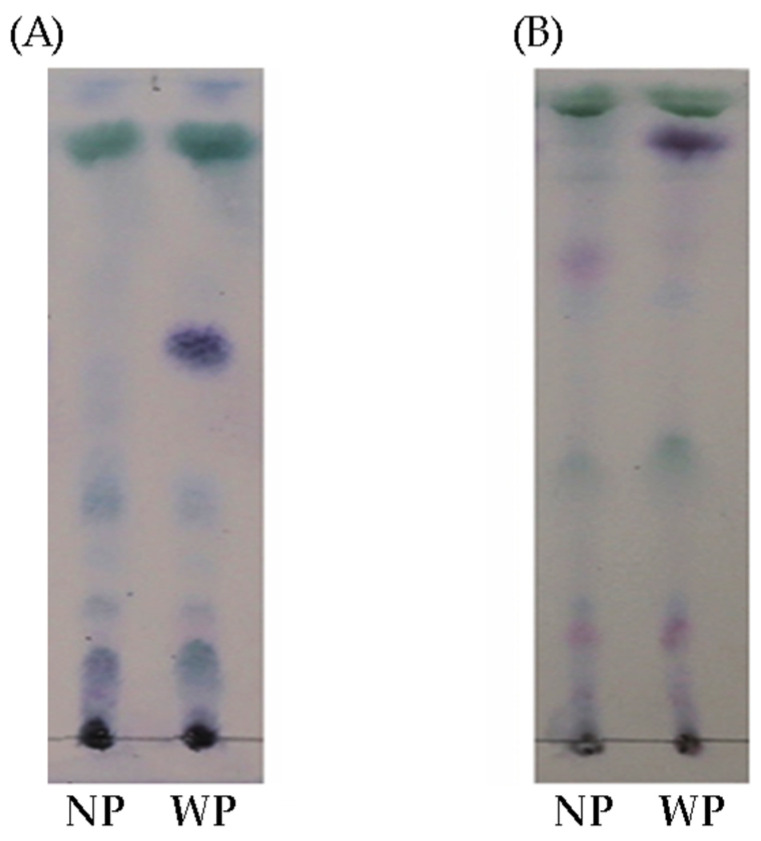
Developed TLC plate photograph of extracts from natural- and wash-processed beans. (**A**) EtOAc/DCM (1:19, *v*/*v*) and (**B**) acetone/DCM (1:19, *v*/*v*). NP and WP stand for the natural and washed processes of coffee beans, respectively.

TLC was used to separate and investigate the phytochemical profile of the natural- and wash-processed coffee bean extracts. Various solvents were tried, and the most favorable solvents (DCM, ethyl acetate, and acetone) were mixed in suitable ratios to generate well-defined substance zones (Figure 4). Achieving a satisfactory elution of the coffee bean extracts from the starting line required a combination of acetone and DCM. TLC plates were further subjected to *p*-anisaldehyde dying (Figure 4). Under both conditions, TLC profiles exhibited wide ranges of hydrophobicity.

The developed TLC fingerprinting showed dominantly five bands and six bands in natural and processed beans, respectively. The main difference between the natural- and wash-processed samples was found as a purple spot at *R_f_* value at 0.55 (Figure 4A) and 0.88 (Figure 4B).

### 3.5. Untargeted Metabolomics Analysis

**Figure 5:**
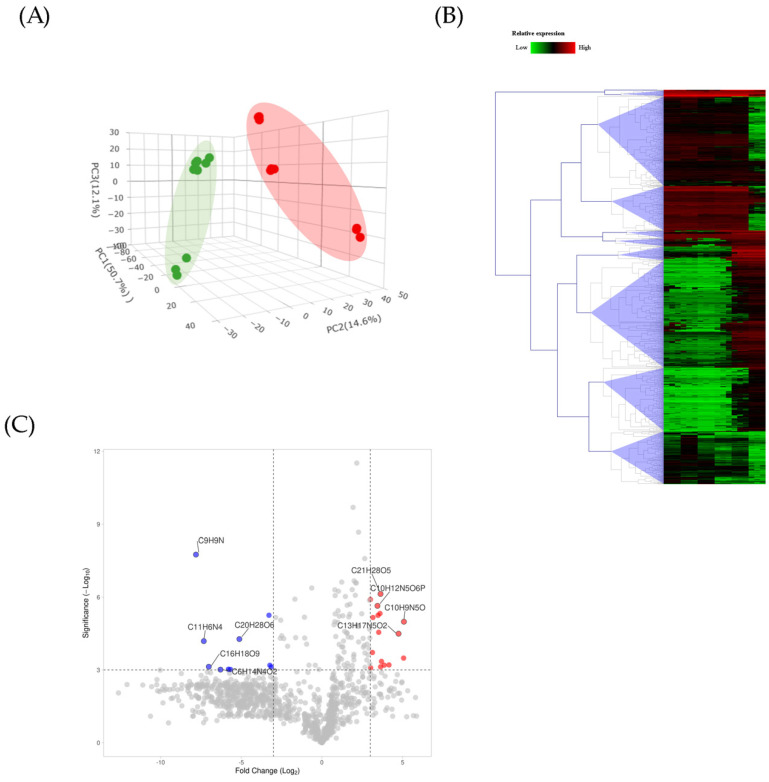
Metabolomics profiling. (**A**) 3D diagram of PCA of two processed beans methods. (**B**) Volcano plots were obtained from all annotated compounds. The threshold change was set as 3.0. The red and blue dots are the metabolites presented at significantly different amounts in the beans processed by natural method compared to washed method. Grey dots indicate metabolites not significantly influenced between the beans processed by natural method compared to washed method. (**C**) Heat map analysis between natural and washed methods. Red-filled and green-colored dots indicate increased and decreased levels of their abundance, respectively.

The untargeted metabolic method is a very sensitive method for the identification and quantification of metabolites. The data reproducibility using the TLC of all LC injections is shown in Supplementary Data S1. Relatively abundant compounds among 18 LC runs (three biological and three instrumental replicates) are considered differentially expressed. In total, 1114 compounds were successfully annotated with stereoisomeric configurations. Based on the database, 603 compounds were identified with complete annotation (Supplementary Data S2). To investigate the variability of the differential metabolome profiles, 3D-PCA analysis was performed by comparing the two sample groups in the 18 LC runs and evaluating intra- and intervariations between the beans processed by natural and washed methods. The PCA of three independent biological replicates and three technical replicates of each group was analyzed. The PCA results demonstrated differences in the metabolome profiles (Figure 5A). The PCA diagram shows that the total score of the first three PCs is 50.7%, representing the most information from the LC-MS original identifier. As shown in Figure 5A, the distribution area of the beans processed by natural and washed methods indicated the clear agglomeration for the sample in the same method without any overlap. These results indicate the beans processed with natural and washed methods exhibited differences in their metabolome profiles.

To further explore the quantitative metabolome data, the 1114 compounds that altered their abundance were visualized using hierarchically clustered heat maps and a volcano plot to discern the dissimilarity between the beans processed by natural and washed methods (Figure 5B,C). The completed annotated metabolites are shown in Supplementary Data S2. As shown in Figure 5B, a comprehensive cluster analysis was conducted on 18 LC runs (n = 18), and differential metabolites were determined. The volcano plot was used to compare the beans processed by natural and washed methods (log_2_ fold change (FC) and −log *p*-value) with an FC threshold of 3 and −log(*p*-value) > 3. Each point represents an annotated chemical formula of a metabolite. The volcano plot allowed us to display any large magnitude changes that are also statistically significant for ranking. Among the identified differential annotated metabolites, a total of 13 amino acids, 7 dipeptides (GV, LP, GL, LL, LF, CG, and GC), and 6 tripeptides (DPH, WQH, KKK, EKK, glutathione) were identified and are shown in Table 2.

### 3.6. Quantification of CGA

**Figure 6:**
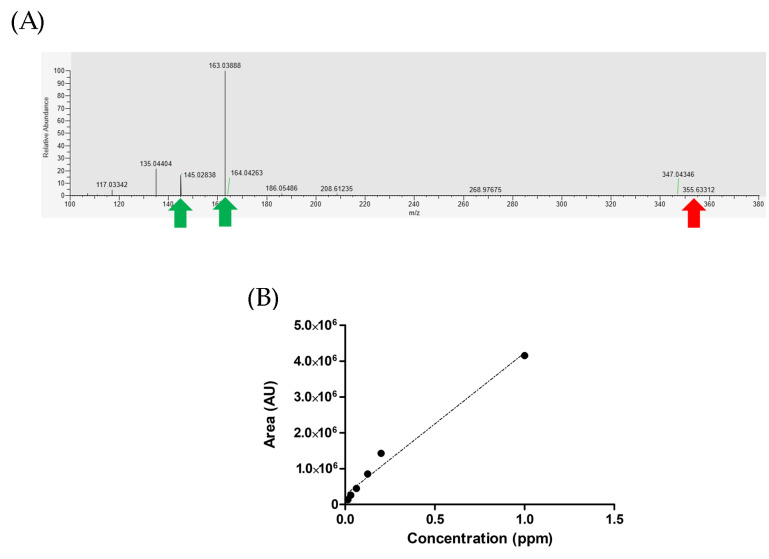
Characteristic of CGA in absolute quantification using LC-MS/MS. (**A**) Fragmentation mass spectra by higher energy collisional dissociation (HCD) of standard compounds. (**B**) A calibration curve was generated for CGA (0.016–1 ppm).

Standard CGA optimization was performed in positive mode LC-MS/MS. A fragmentation mass spectra (MS2) pattern was required to support the appropriate PRM for CGA (Figure 6A). The MS1 and MS2 quality of CGA is shown in Supplementary Data S1. As shown in Figure 5A, there is a minor peak of CGA at 335.63312 (red arrow) and its derivative fragment ions (163.03888 and 145.02838; green arrow). Within the CGA concentration range of 0.016–1 ppm, the area showed a linear regression with an R-square value of 0.9811, as depicted in Figure 6B.

In 0.1 mg dried weight of coffee beans, the washed process of coffee beans contains an average of 1.243 ± 0.151 μg of CGA, while the natural process of the coffee beans contains an average of 1.375 ± 0.192 μg of CGA. A comparison between the washed and natural processes of coffee beans revealed no significant difference in CGA levels.

## 4. Discussion

There have been several studies that have investigated the potential health benefits of coffee for people with T2DM. More research is crucially needed to fully understand the relationship between the compounds within coffee and T2DM. According to enzyme bioactivity testing, beans processed using the natural method significantly inhibited DPP4 and α-amylase functions. From a pharmaceutical aspect, these inhibition values showed a slight (10–20%) inhibition level. However, from a functional food aspect, coffee, as a beverage, is not a medicine or drug. For this reason, it is impossible to have great potential as a known drug. In addition, the slowing down of starch digestion by coffee extract is not dependent on decreasing enzymatic activity alone; the coffee extract is affected by perturbing the binding affinity at active sites of enzymes. There were reports that in moderate coffee consumption, the compounds in coffee extract can form complexes with α-amylase via a static quenching mechanism [23]. From another perspective, drinking coffee may be beneficial for developing T2DM in people who already have the condition by, in part, the presence of dominant compounds such as chlorogenic acids [24]. This suggested to us that the lower inhibition level may not reflect the realistic situation in our body. In addition, our coffee beans with a specific geographical origin and processing could be developed into a beverage with health benefits in term of health prevention and maintenance.

To screen the metabolites extracted from natural- and wash-processed beans, TLC profiling was performed. After developing with mobile phases (Table 1) and dying with *p*-anisaldehyde reagent, prominent purple spots were observed in the wash-processed bean extract but not found in the natural-processed bean extract. Furthermore, to identify the diverse metabolites present in the extract, LC-MS/MS analysis was performed. LC-MS/MS is the most acceptable approach for the identification of both polar and nonpolar metabolites. Our findings found differences in the metabolome profiling of processed coffee beans using natural and washed methods. As shown in Figure 5A, the beans processed by natural methods are grouped in different regions in the XYZ coordinates. The sampling set near the group was highly correlated in terms of the metabolome profiling of each group, whereas the sample set distance was less correlated. The intragroup variations were slightly smaller than the intergroup variations, which may be due to the small sample size or largely uncontrollable factors, such as processing time variation and other factors pre- and postsample collection. Clustering using a hierarchical algorithm can visually demonstrate the difference in the metabolome in different processed beans. From a sensory perspective, organic acids and amino acids are the most important components of coffee. The amino acid profiling abundance is shown in Table 2. It comprises a large fraction of the total mass, as much as 11% of the green and 6% of the roasted beans [25]. The organic acids represent a large proportion of the total dried mass, with up to 11% of green coffee beans and 6% of roasted coffee beans [26]. The amount of specific organic acids in coffee beans in roasted beans greatly influences the quality of the final cup [27]. The most prominent components of green coffee are citric acid, malic acid, and quinic acid. Increasing any amount of organic acid will reduce the pH and increase titratable acidity. A deeper insight into the metabolome profiling among the various compounds of amino acids, along with the outstanding presence of seven dipeptides (GV, LP, GL, LL, LF, CG, and GC) and seven tripeptides (DPH, WQH, DPH, KKK, EKK, and glutathione), in coffee beans has never been described.

Owing to the favorable attributes associated with short peptides, they exhibit superior characteristics in relation to metabolism, absorption, and safety when contrasted with a range of drug-like compounds [28,29]. The evidence showed the correlation between short peptides and DPP4 activity [30,31]. In the BIOPEP-UWM database, it reported seven dipeptides, including QY, NY, EY, DR, EK, VL, and VG, reported as effective DPP-IV inhibitory peptides [32]. In the context of structure–bioactivity relationships, DPP4 inhibitory peptides exhibit a preference for hydrophobic residues, notably valine, alanine, leucine, isoleucine, methionine, phenylalanine, tryptophan, and tyrosine, at their N-terminal region [31,33]. Notably, our findings revealed that six out of seven di-peptides displayed hydrophobic amino acids at their N-terminal regions, aligning with previous research. This observation suggests the potential for these peptides to bind to the binding sites of DPP4 and inhibit its functions. However, in order to confirm the inhibition of DPP4, it is necessary to validate the inhibitory effect and understand its mechanism using synthetic peptides.

In addition, we found high level of leucine, methionine, and tyrosine in beans processed with the washed method compared to the natural method. As the coffee beans were controlled in terms of the type of coffee plant and the growing conditions, they are only different in terms of the coffee processing method. Therefore, we suggest that the beans processed by natural and washed methods exhibited different short peptide and amino acid contents. Amino acids and organic acids are important aroma precursors in roasting process which will impact the coffee beverage final cup quality.

The previous evidence also suggested that naturally processed beans tend to have a higher concentration of volatile compounds than washed processed beans [20]. However, our result found 1,2,4-trimethoxybenzene in washed processed beans at a significantly higher level than the naturally processed beans (*p*-value = 3.3 × 10^−3^). In terms of this contrary trend, we suggest that the natural and washed processed beans may be different in terms of processing steps, times, and equipment. In addition, an abundance of 1,2,4-trimethoxybenzene, which is the flavor of coffee, can vary depending on a number of factors, including the type of coffee bean, area, and the conditions under which the beans are harvested. These uncontrollable factors may contribute to the 1,2,4-trimethoxybenzene in washed processed beans exhibiting a higher level than in natural processed beans.

As mentioned above, the metabolome of processed beans collected from natural and washed methods may be obviously different. However, this study was limited to representing the overall differences between coffee beans samples. Different metabolites in coffee beans do not correspond to different nutritional values, aromas, or tastes. Moreover, the coffee beans should be collected with more biological replicates for the improvement of the accuracy of future research.

We utilized a known metabolite library to obtain accurate and reliable results. By comparing the unknown feature spectrum with its closest matches in the mzCloud library, we identified similarities in LC-MS spectrum information and characteristic fragmentation ions. In the MS2 spectrum of CGA, we observed distinct peaks at 163.03888 and 145.02838 (indicated by green arrows in Figure 6A), corresponding to C_9_H_7_O_3_^+^ and C_9_H_5_O_2_^+^ ions, respectively. These findings are consistent with the information present in the database, confirming the reliability and accuracy of the CGA content in our experiment.

## 5. Conclusions

Our study examined how natural and wet coffee processing methods impact coffee bean phytochemical compositions using TLC- and LC-MS/MS-based metabolomic approaches. We discovered that these processing methods significantly alter phytochemical profiles, potentially affecting their bioactivity, especially in relation to antidiabetic enzyme inhibition. We observed substantial differences in amino acids and short peptides between naturally processed and wet-processed coffee beans. Notably, natural processing exhibited superior inhibition of two key diabetic-related enzymes, α-amylase and DPP4, compared to wet processing. Interestingly, the CGA levels remained consistent between the two processing methods. Our findings highlight the importance of processing methods in shaping coffee bean phytochemicals and their potential in managing diabetes. Further research may yield insights for coffee-based diabetes interventions.

## Figures and Tables

**Table 1:** The ratios of solvents used as mobile phases.

Solvents	Ratio (*v*/*v*)
Ethyl acetate/DCM	1:19
Acetone/DCM	1:19

**Table 2:** Amino acids and short peptide abundance ratio of coffee beans processed by natural and washed methods. The values are the average from 3 biological replications and 3 technical replications (n = 9).

Name	Retention Time (min)	Log_2_ FC	Adj. *p*-Value
Amino acids			
Phenylalanine	5.835	−0.33	<0.001
Valine	1.307	0.2	0.006
Methionine	1.423	0.13	0.009
Histidine	0.8	0.22	0.012
Lysine	0.787	0.21	0.014
Leucine	2.471	0.1	0.015
Tyrosine	1.817	0.14	0.028
Serine	0.88	0.31	0.068
Tryptophan	6.625	−1.25	0.093
Glutamic acid	0.884	3.57	0.223
Asparagine	0.881	1.01	0.420
Arginine	0.873	2.78	0.987
Dipeptides			
LL	5.159	0.26	0.007
LP	4.592	0.17	0.007
LF	5.594	0.30	0.013
GV	1.433	0.16	0.013
GC	1.382	0.33	0.040
CG	1.346	0.28	0.053
GL	2.65	0.26	0.024
Tripeptides			
EKK	6.292	−0.20	<0.001
Glutathione	1.343	0.19	0.019
KKK	5.013	−0.51	0.015
WQH	5.723	0.69	0.019
DPH	9.745	0.18	0.034

## Data Availability

Upon reasonable request, the corresponding author is willing to provide the supplementary data supporting the results of this study.

## Supplementary Materials

The following supporting information can be downloaded at: https://www.mdpi.com/article/10.3390/foods12213893/s1, Supplementary Data S1: Quality control of MS chromatogram and Supplementary Data S2: List of annotated metabolites.
