# Supplementary material for: Potential of Arabica Coffee Beans from Northern Thailand: Exploring Antidiabetic Metabolites through Liquid Chromatography with Tandem Mass Spectrometry (LC-MS/MS) Metabolomic Profiling across Diverse Postharvest Processing Techniques
Source: Foods. 2023 Oct 24;12(21):3893. doi: 10.3390/foods12213893 (PMC10648821; doi:10.3390/foods12213893)
Supplement: Supplementary file 1 [file foods-12-03893-s001.zip › 13oct_supplementary data S2.pdf]

Supplementary data S2

List of annotated metabolites

The identification of metabolites through LC-MS/MS with HCD in a positive mode is conducted. A total of 605 distinct metabolites have been successfully annotated and identified. The table below provides a comprehensive list of all annotations completed in association with the respective databases.

Name	MW (Da)	RT (min)	Area (AU×10^9)	mzCloud	Ratio	Log2FC	Adj. *p*-value
Caffeine	194.1	4.886	50.64	99.7	0.004	-7.98	0.037
Trigonelline	137	0.918	27.10	99.5	0.934	-0.1	0.064
IN00458	162	4.742	19.69		0.001	-9.5	0.012
(1r,3R,4s,5S)-4-{[(2E)-3-(3,4-dihydroxyphenyl)prop-2-enoyl]oxy}-1,3,5-trihydroxycyclohexane-1-carboxylic acid	354.1	4.737	6.83	61.9	1.040	0.06	0.127
Chlorogenic acid	354.1	4.737	6.54		0.002	-8.7	0.008
(1R,3R,4S,5R)-3,4-bis({[(2E)-3-(3,4-dihydroxyphenyl)prop-2-enoyl]oxy})-1,5-dihydroxycyclohexane-1-carboxylic acid	498.1	5.821	5.07	92	0.820	-0.29	0.205
7-Hydroxycoumarine	162	4.346	4.12	60	0.932	-0.1	0.396
N-[(2E)-3-(3,4-Dihydroxyphenyl)-2-propenoyl]tryptophan	366.1	6.624	3.40		0.000	-12.64	0.017
1,3,5-trihydroxy-4-{[(2E)-3-(3-hydroxy-4-methoxyphenyl)prop-2-enoyl]oxy}cyclohexane-1-carboxylic acid	368.1	5.326	1.98	63.1	0.000	-11.24	0.011
Choline	103.1	0.869	1.79	95.8	0.002	-9.32	0.010
trans-3-Indoleacrylic acid	187.1	4.533	1.36	94.3	0.002	-9.23	0.014
Boldione	284.2	5.536	1.23		0.882	-0.18	0.045
Diprogulic Acid	274.1	3.022	1.15		1.218	0.28	0.118
(1S,3R,4S,5R)-3,5-bis({[(2E)-3-(3,4-dihydroxyphenyl)prop-2-enoyl]oxy})-1,4-dihydroxycyclohexane-1-carboxylic acid	516.1	5.822	1.13	70.7	0.005	-7.68	0.027
Cnidioside A	390.1	5.328	1.09	95.5	0.995	-0.01	0.692
Crocetin	328.2	5.536	1.07		0.932	-0.1	0.303
trans-Zeatin	219.1	2.038	1.06	62.5	0.961	-0.06	0.158
Phthaldialdehyde	134	4.742	1.01		0.974	-0.04	0.239
1-(3-methoxy-4-{[(2S,3R,4S,5S,6R)-3,4,5-trihydroxy-6-({[(2R,3R,4R,5R,6S)-3,4,5-trihydroxy-6-methyloxan-2-yl]oxy}methyl)oxan-2-yl]oxy}phenyl)ethan-1-one	512.1	6.274	0.99	62.2	0.688	-0.54	0.200
D-(+)-Maltose	364.1	1.011	0.96	98.3	1.185	0.25	0.118
ortho-Phthalaldehyde	134	4.743	0.93		0.003	-8.28	0.016
Coumarin	146	5.135	0.72	65.6	1.148	0.2	0.143
(1S,4aS,7aS)-7-({[(2E)-3-phenylprop-2-enoyl]oxy}methyl)-1-{[(2S,3R,4S,5S,6R)-3,4,5-trihydroxy-6-(hydroxymethyl)oxan-2-yl]oxy}-1H,4aH,5H,7aH-cyclopenta[c]pyran-4-carboxylic acid	542.1	0.972	0.68	77.3	0.013	-6.24	0.016
(2E)-3-(3,4-dimethoxyphenyl)prop-2-enoic acid	190.1	6.82	0.57	97.1	1.318	0.4	0.116
Leu-Phe	278.2	5.594	0.01		1.23	0.30	0.013
Aspirin	180	4.799	0.42		0.005	-7.52	0.015
hymecromone	176	6.369	0.38		0.001	-9.65	0.013
Furfural	96.02	0.956	0.37		0.023	-5.46	0.010
L-Glutamic acid	147.1	0.884	0.37	99	11.88	3.57	0.223
Adenosine	267.1	1.9	0.36	99.6	0.935	-0.1	0.122
6-Methylquinoline	143.1	4.831	0.33	89.7	0.024	-5.39	0.021
Vorinostat	264.1	4.67	0.33		0.001	-10.6	0.007

Name	MW (Da)	RT (min)	Area (AU×10⁹)	mzCloud	Ratio	Log2FC	Adj. *p*-value
Indole-3-acetaldehyde	159.1	2.698	0.30		0.001	-10.15	0.014
N-Acetyl-L-phenylalanine	207.1	4.639	0.29		0.973	-0.04	0.075
Nicotinamide	122	1.337	0.29	95.3	6.993	2.8	0.000
Choline Alfoscerate	257.1	0.883	0.28		0.002	-8.66	0.018
DL-Stachydrine	143.1	1.144	0.28	94.7	0.002	-8.66	0.015
Phloroglucinol	126	0.962	0.26	74	1.057	0.08	0.122
Stachydrine	143.1	1.287	0.26		0.001	-9.82	0.020
8-Methyl-3-oxo-8-azoniabicyclo[3.2.1]octane	140.1	18.12	0.26		0.994	-0.01	0.627
Hexamine	140.1	18.12	0.25		0.001	-9.81	0.013
cipamfylline	275.1	2.627	0.25		38.462	5.28	0.002
Kojic acid	142	2.013	0.25	99.6	0.789	-0.34	0.196
L-Tyrosine	181.1	1.817	0.25	94.9	1.10	0.14	0.028
meturedepa	275.1	2.439	0.24		33.333	5.07	0.001
LV1850000	140	5.523	0.24		0.046	-4.45	0.019
Isoniazid	137.1	4.887	0.24		1.032	0.05	0.668
5-methyl-4-{[(2S,3R,4S,5S,6R)-3,4,5-trihydroxy-6-(hydroxymethyl)oxan-2-yl]oxy}-2H-chromen-2-one	338.1	5.134	0.23	67.8	1.225	0.29	0.131
NP-022035	538.1	5.806	0.22	96.9	0.845	-0.24	0.726
MUD	338.1	5.134	0.22		0.010	-6.65	0.047
Vedaprofen	282.2	5.535	0.22		0.955	-0.07	0.057
(2S,3S,4S,8R,9S,13R,14R,15R,16R)-3,4,8,14,15-pentahydroxy-2,13,16-trimethyl-6-methylidene-10-oxatetracyclo[7.6.1.0Â?,â◎·.0ÂaÂ?,Âaâ◎¶]hexadecan-11-one	406.1	3.93	0.21	88.8	0.109	-3.2	0.036
NP-016928	298.2	5.361	0.21	86.1	1.034	0.05	0.800
7-Hexopyranosyl-N-(3-methyl-2-buten-1-yl)-7H-purin-6-amine	365.2	5.185	0.21		0.014	-6.16	0.027
17α-Ethynylestradiol	296.2	9.64	0.21	89.4	2.169	1.12	0.045
SKF-97,541	137.1	4.884	0.21		1.052	0.07	0.117
D-(+)-Pyroglutamic Acid	129	0.883	0.20	99.1	0.746	-0.42	0.466
5,7-dihydroxy-2-(3-hydroxy-4-methoxyphenyl)-3,6-dimethoxy-4H-chromen-4-one	360.1	5.137	0.20	91.3	0.929	-0.11	0.147
LU3453000	84.02	0.955	0.19		0.012	-6.41	0.014
Embelin	294.2	9.743	0.19		0.002	-8.69	0.015
D-(+)-Proline	115.1	0.959	0.18	99.8	1.486	0.57	0.121
2-Pyrrolidone	85.05	0.888	0.17		0.018	-5.8	0.009
QV1MVO1R	209.1	3.813	0.17		0.659	-0.6	0.382
(2S)-8-[(1E)-3-hydroxy-3-methylbut-1-en-1-yl]-5,7-dimethoxy-2-phenyl-3,4-dihydro-2H-1-benzopyran-4-one	406.1	2.469	0.17	88.2	1.919	0.94	0.059
3477	240.1	0.898	0.17		0.007	-7.12	0.016
Dichloromethane	83.95	0.755	0.17		1.942	0.96	0.035
Dehydroepiandrosterone (DHEA)	270.2	5.359	0.17	89	0.829	-0.27	0.369
Theobromine	180.1	4.12	0.16	99.3	0.712	-0.49	0.110
tin(ii) hydride	121.9	0.828	0.16		0.889	-0.17	0.006
Benzenol	94.04	1.817	0.16		0.002	-8.89	0.011
5-Fluoroindole-2-carboxylic acid	179	18.17	0.15		1.045	0.06	0.127
Gibberellin A12 aldehyde	316.2	5.353	0.15		0.012	-6.44	0.000
NP-014175	316.2	5.357	0.15	82.5	0.829	-0.27	0.285
(1S,3R,4R,5R)-1,3,4-trihydroxy-5-{[(2E)-3-(4-hydroxy-3-methoxyphenyl)prop-2-enoyl]oxy}cyclohexane-1-carboxylic acid	406.1	5.331	0.15	91.3	1.005	0.01	0.148

Name	MW (Da)	RT (min)	Area (AU×10^9)	mzCloud	Ratio	Log2FC	Adj. *p*-value
2-Anisic acid	134	5.812	0.15	61.3	0.009	-6.77	0.035
Leucylproline	228.1	4.592	0.14	84.2	1.13	0.17	0.007
MFCD01318772	183.1	0.821	0.14		0.002	-9.11	0.024
3-{[2-({[2,3-dihydroxy-2-(1-hydroxyethyl)butanoyl]oxy}methyl)phenyl]carbamoyl}propanoic acid	391.1	4.638	0.14	66.5	1.289	0.37	0.112
FG7175000	145.1	4.532	0.14		0.336	-1.57	0.022
1-Methyl-1,2,3,4-tetrahydro-Î^{2}-carboline-3-carboxylic acid	230.1	5.161	0.13		0.028	-5.16	0.015
(2E)-3-(2-{[(2S,3R,4S,5S,6R)-3,4,5-trihydroxy-6-(hydroxymethyl)oxan-2-yl]oxy}phenyl)prop-2-enoic acid	308.1	6.353	0.13	69.7	1.203	0.27	0.120
2908	85.09	2.258	0.13		0.001	-10.01	0.006
Amoxicillin	365.1	1.112	0.12		1.060	0.09	0.160
N-Methylpyrrolidone	99.07	3.982	0.12		0.001	-10.18	0.008
gibberellin A24	346.2	6.212	0.12		0.008	-6.89	0.012
8-Hydroxyquinoline	145.1	4.525	0.12	61	0.387	-1.37	0.989
Pipecolic acid	129.1	1.305	0.12	97.1	0.001	-9.39	0.007
NP-019811	107	1.008	0.12	95.9	1.522	0.61	0.085
2-Aminoethylarsonate	169	18.42	0.11		1.225	0.29	0.183
Indole	117.1	4.558	0.11	79.2	0.002	-8.9	0.006
Diethylpyrocarbonate	162.1	0.961	0.11		0.156	-2.68	0.010
7-(2-hydroxypropan-2-yl)-1,4a-dimethyl-1,2,3,4,4a,9,10,10a-octahydrophenanthrene-1-carboxylic acid	298.2	5.646	0.11	76.5	1.923	0.94	0.107
Gonan-3-yl hydrogen sulfate	328.2	7.239	0.11		1.534	0.62	0.117
NP-005196	264.1	4.653	0.11	62.6	0.119	-3.07	0.016
Dimethyl fumarate	144	0.958	0.11		0.071	-3.81	0.006
D-Raffinose	526.2	0.976	0.11	80	1.227	0.29	0.221
3-(3,4-dihydroxyphenyl)propanoic acid	164	1.785	0.10	79.9	0.874	-0.19	0.159
(2S,3R,4S,5S,6R)-3,4,5-trihydroxy-6-{[4-(2-hydroxypropan-2-yl)cyclohex-1-ene-1-carbonyloxy]methyl}oxan-2-yl 4-(2-hydroxypropan-2-yl)cyclohex-1-ene-1-carboxylate	534.2	4.917	0.10	76	1.034	0.05	0.171
Leucine	131.1	2.471	0.10	76.2	1.07	0.1	0.015
Kinetin	215.1	0.887	0.10		32.258	5.01	0.000
Oxibendazole	249.1	9.684	0.10		0.004	-8.07	0.073
3,4,5-trihydroxy-6-(hydroxymethyl)oxan-2-yl 2-(prop-1-en-2-yl)-2,3-dihydro-1-benzofuran-5-carboxylate	388.1	6.631	0.10	84.6	0.004	-7.82	0.008
Î^{3}-Oxo-3-pyridinebutanal	163.1	3.814	0.10		0.703	-0.51	0.171
Caffeic acid	180	5.964	0.10	87.1	1.094	0.13	0.150
2-Acetamidophenol	151.1	1.349	0.09		1.222	0.29	0.124
NP-004022	392.1	2.49	0.09	76.7	0.499	-1	0.133
NP-021085	300.2	5.535	0.09	73.4	0.943	-0.09	0.027
Troxipide	294.2	3.885	0.09		0.002	-8.83	0.011
L-Histidinol phosphate	221.1	0.817	0.09		0.677	-0.56	0.003
1,4-Dimethyl-5H-pyrido[4,3-b]indol-3-amine	211.1	5.506	0.09		0.007	-7.19	0.015
Acetophenone	120.1	1.96	0.09	71.8	0.005	-7.78	0.031
Paracetamol	151.1	1.351	0.08		0.004	-8.12	0.004
Prolylleucine	228.1	1.509	0.08	93.9	1.014	0.02	0.562
Coumarone	118	5.135	0.08		0.005	-7.64	0.010
Adenosine 5'-monophosphate	347.1	1	0.08	99.4	0.442	-1.18	0.997

Name	MW (Da)	RT (min)	Area (AU×10^9)	mzCloud	Ratio	Log2FC	Adj. *p*-value
methyl 5-methoxy-2-{[(2S,3R,4S,5S,6R)-3,4,5-trihydroxy-6-({[(2S,3R,4S,5R)-3,4,5-trihydroxyoxan-2-yl]oxy}methyl)oxan-2-yl]oxy}benzoate	498.1	4.989	0.08	93.1	9.804	3.29	0.300
Mitragynine	398.2	7.022	0.08	98.8	2.198	1.14	0.139
(2R,3S,4S,5R,6S)-2-({[(2S,3R,4R)-3,4-dihydroxy-4-(hydroxymethyl)oxolan-2-yl]oxy}methyl)-6-(3,4,5-trimethoxyphenoxy)oxane-3,4,5-triol	516.1	4.742	0.08	65.9	1.018	0.03	0.159
[(2R,2'S,4'aS,5'R,5''S,6'R,8'aS)-2'-(acetyloxy)-5''-(furan-3-yl)-6'-methyl-2'',8'-dioxo-octahydrodispiro[oxirane-2,1'-naphthalene-5',3''-oxolane]-8'a-yl]methyl acetate	498.1	4.991	0.08	94.9	0.048	-4.4	0.032
NP-022216	552.1	6.381	0.08	96.2	0.984	-0.02	0.208
Adenine	135.1	1.016	0.08	99.2	3.484	1.8	0.023
NP-013296	460.2	4.453	0.08	87.6	1.172	0.23	0.347
Estriol	310.2	6.038	0.08	81.6	3.876	1.95	0.004
NP-019150	446.1	4.51	0.07	92.7	1.202	0.27	0.126
benzoquinone	108	0.959	0.07		0.041	-4.62	0.011
Benzyl Î²-primeveroside	402.2	4.949	0.07		0.997	0	0.136
NP-008993	336.2	9.778	0.07	99.8	23.256	4.53	0.002
5,7-dihydroxy-3-(4-hydroxyphenyl)-6-methoxy-4H-chromen-4-one	322.1	4.736	0.07	78.8	1.136	0.18	0.175
1,2,3,4-Tetrahydro-Î²-carboline-3-carboxylic acid	216.1	5.019	0.07		0.375	-1.41	0.002
9-hydroxy-7-(2-hydroxypropan-2-yl)-1,4a-dimethyl-1,2,3,4,4a,9,10,10a-octahydrophenanthrene-1-carboxylic acid	314.2	6.03	0.07	71.8	2.155	1.11	0.065
DL-Tryptophan	204.1	6.625	0.06	98.7	0.42	-1.25	0.093
20,21-dihydroxy-10-methoxy-1,4,14,19,19-pentamethyl-2,7,18-trioxapentacyclo[11.9.0.0Âl,ÂaÂa.0â©µ,â©a.0Âaâ©´,Â?â©°]docosa-3,5(9),10-triene-8,17-dione	482.2	4.397	0.06	64.9	2.874	1.52	0.057
Deferiprone	139.1	0.994	0.06		0.964	-0.05	0.362
L-(+)-Leucine	131.1	2.258	0.06		0.002	-8.98	0.020
1,5-DAN	158.1	6.623	0.06		0.914	-0.13	0.299
Sinapinic acid	206.1	6.3	0.06	91.6	0.980	-0.03	0.160
[(2R,3S,4S,5R,6R)-6-[2-(3,4-dihydroxyphenyl)ethoxy]-3,4,5-trihydroxyoxan-2-yl]methyl (2E)-3-(3,4-dihydroxyphenyl)prop-2-enoate	500.1	6.132	0.06	67.9	0.810	-0.3	0.209
NP-000294	234.2	11.15	0.06	87.9	0.002	-9.05	0.077
4-hydroxy-7-methyl-2-(2-{[3,4,5-trihydroxy-6-(hydroxymethyl)oxan-2-yl]oxy}propan-2-yl)-2H,3H,5H-furo[3,2-g]chromen-5-one	460.1	6.761	0.06	96.4	0.982	-0.03	0.145
DL-Arginine	174.1	0.873	0.06	98	6.87	2.78	0.987
Butylparaben	194.1	9.857	0.06		1.222	0.29	0.117
Ethyl paraben	166.1	9.856	0.06		1.215	0.28	0.117
(2S,3S,4S,5R,6R)-6-[3-(benzoyloxy)-2-hydroxypropoxy]-3,4,5-trihydroxyoxane-2-carboxylic acid	394.1	3.745	0.06	92.5	0.855	-0.23	0.343
Dimethyl (3-oxocyclohexyl)malonate	228.1	6.212	0.06		0.004	-7.81	0.007
NP-007585	322	4.735	0.06	77.6	0.039	-4.69	0.028
Dimethylphenylpiperazinium	191.2	1.324	0.06		0.948	-0.08	0.123
L-Theanine	174.1	0.997	0.06		0.025	-5.3	0.138
Paraxanthine	180.1	4.428	0.05	98.8	0.767	-0.38	0.116
4-Hydroxybenzaldehyde	122	1.775	0.05	98.8	0.856	-0.22	0.303
Uracil	112	1.623	0.05	98.9	1.821	0.87	0.096
Hexanenitrile	97.09	1.287	0.05		0.003	-8.35	0.015
NP-007909	206.1	5.232	0.05	82.4	1.045	0.06	0.682
(1S,4S,5R,9R,13S)-5,9-dimethyl-14-methylidenetetracyclo[11.2.1.0Âa,Âaâ©°.0â©´,â©a]hexadec-10-ene-5-carboxylic acid	300.2	6.19	0.05	74.7	1.852	0.89	0.113
Allantoin	158	0.756	0.05		1.241	0.31	0.130

Name	MW (Da)	RT (min)	Area (AU×10^9)	mzCloud	Ratio	Log2FC	Adj. *p*-value
Biocytin	372.2	4.889	0.05	69.7	1.276	0.35	0.112
Guanine	151.1	2.276	0.05	97.9	2.101	1.07	0.090
Pyroquilon	173.1	3.754	0.05		71.429	6.12	0.001
1-(4-{[(2S,3R,4S,5S,6R)-3,4,5-trihydroxy-6-({[(2S,3R,4S,5R)-3,4,5-trihydroxyoxan-2-yl]oxy}methyl)oxan-2-yl]oxy}phenyl)ethan-1-one	452.1	3.835	0.05	99.1	0.984	-0.02	0.157
5-Hydroxy-DL-tryptophan	220.1	2.743	0.05		0.002	-8.69	0.010
Veronal	184.1	1.002	0.05		0.056	-4.16	0.014
methyl 2-ethyl-4-[(3R,4R,5S)-5-hydroxy-4,5-dimethyl-2-oxooxolan-3-yl]-2-methyl-3-oxobutanoate	308.1	8.752	0.05	96.2	0.441	-1.18	0.145
(2R,7S,13R,14R,16S,19R,20S)-19-(furan-3-yl)-9,9,13,20-tetramethyl-5,12,17-trioxo-4,8,15,18-tetraoxahexacyclo[11.9.0.0Â?,â◎·.0Â?,Âaâ◎°.0Âaâ◎´,Âaâ◎¶.0Âaâ◎´,Â?â◎°]docosan-11-yl acetate	566.1	6.831	0.05	91	1.112	0.15	0.118
Ethylparaben	166.1	9.854	0.05		0.002	-8.9	0.015
trimethadione	143.1	0.978	0.05		0.079	-3.67	0.019
2-O-ETHYL ASCORBIC ACID	204.1	0.879	0.05		3.636	1.86	0.008
1,7-Dimethyl-1,3,7-cyclodecatriene	162.1	4.729	0.05		3.584	1.84	0.106
cyclandelate	276.2	8.721	0.05		0.003	-8.4	0.012
Nicotinic acid	123	1.324	0.05	99.6	1.125	0.17	0.126
4-Methoxycinnamic acid	160.1	5.209	0.05	88.8	1.209	0.27	0.074
NP-020713	330.2	6.881	0.05	81	6.173	2.63	0.015
1-Methyladenine	149.1	1.006	0.05	99.7	0.992	-0.01	0.179
Oxagrelate	276.1	4.959	0.05		0.068	-3.87	0.020
(2R,3R,4S,5S,6R)-2-[2-(4-methoxyphenyl)ethoxy]-6-({[(2S,3R,4S,5S)-3,4,5-trihydroxyoxan-2-yl]oxy}methyl)oxane-3,4,5-triol	468.2	5.198	0.05	96.5	1.259	0.33	0.135
Biacetyl	86.04	0.884	0.04		0.063	-3.99	0.019
uredepa	219.1	0.882	0.04		15.873	3.98	0.000
2-Oxo-3-(phosphonatooxy)propanoate	181	0.845	0.04		0.755	-0.41	0.297
NP-002676	308.1	5.208	0.04	82.2	1.155	0.21	0.073
Varespladib	380.1	7.052	0.04		0.011	-6.51	0.010
2,6-Dimethylquinoline	157.1	5.163	0.04		0.361	-1.47	0.047
Estrone	292.1	6.036	0.04	85.4	9.709	3.27	0.007
Androsterone	272.2	6.191	0.04	88.8	1.992	0.99	0.112
tolimidone	202.1	2.747	0.04		0.003	-8.19	0.007
5-Hydroxymethyl-2-furaldehyde	126	6.215	0.04		1.667	0.74	0.136
Kaempferol	286.1	5.82	0.04	99.2	1.011	0.02	0.150
Indoleacetylaspartate	290.1	5.814	0.04		0.012	-6.41	0.013
7-Isopropyl-4a-methyl-1-methylene-1,2,3,4,4a,9,10,10a-octahydrophenanthrene	254.2	6.193	0.04		0.013	-6.23	0.004
NP-012228	318.1	5.82	0.04	61.4	0.833	-0.26	0.227
Methylarsonic acid	139.9	18.21	0.04		1.342	0.43	0.089
NP-011220	210.1	5.675	0.04	96.5	10.417	3.38	0.006
NP-020521	278.2	11.2	0.04	96.9	2.457	1.3	0.034
Cysteinylglycine	178	1.346	0.04	95.9	1.21	0.28	0.053
(12Z)-9,10,11-trihydroxyoctadec-12-enoic acid	352.2	7.718	0.04	98.1	4.545	2.18	0.015
KG7537500	302.2	5.535	0.04		0.011	-6.57	0.011
N-Acetylornithine	174.1	0.996	0.04		0.687	-0.54	0.268

Name	MW (Da)	RT (min)	Area (AU×10^9)	mzCloud	Ratio	Log2FC	Adj. *p*-value
Nafenopin	310.2	5.686	0.04		0.007	-7.08	0.007
Crotonic acid	86.04	0.88	0.04	65.9	2.525	1.33	0.012
NP-014287	278.2	9.775	0.04	91.4	27.027	4.77	0.001
NP-020014	292.1	5.686	0.04	81	1.332	0.41	0.130
Isoquinoline	129.1	5.519	0.04	99.2	0.631	-0.66	0.494
glycidyl oleate	338.3	14.7	0.04		1.074	0.1	0.127
NP-020078	302.2	5.535	0.04	62.3	0.900	-0.15	0.382
Pyridoxal	167.1	1.361	0.04	79.2	52.632	5.73	0.003
Acitretin	326.2	5.85	0.04		0.029	-5.11	0.013
NP-014693	334.2	8.721	0.03	63.5	2.882	1.53	0.068
L-serine phosphoethanolamine	228.1	3.232	0.03		0.003	-8.27	0.011
tretamine	204.1	0.978	0.03		0.791	-0.34	0.806
NP-022473	344.2	4.42	0.03	81.9	1.209	0.27	0.050
NP-012028	570.2	4.945	0.03	97.5	0.101	-3.31	0.004
(7E,13E)-9,15-dihydroxy-4,10,16-trimethyl-1,5,11-trioxacyclohexadeca-7,13-diene-2,6,12-trione	364.1	6.632	0.03	68.3	0.018	-5.77	0.036
FENAMIC ACID	213.1	5.161	0.03		0.132	-2.92	0.037
5-hydroxy-6,8-dimethoxy-7-{[(2S,3R,4S,5S,6R)-3,4,5-trihydroxy-6-(hydroxymethyl)oxan-2-yl]oxy}-2H-chromen-2-one	422.1	2.461	0.03	97.5	1.328	0.41	0.092
NP-000124	250.1	5.267	0.03	94.5	0.306	-1.71	0.483
1-(beta-D-ribofuranosyl)thymine	258.1	1.938	0.03		0.005	-7.54	0.011
Amide C18	283.3	14.75	0.03		0.012	-6.37	0.009
HIAA	191.1	3.824	0.03		0.003	-8.52	0.040
2-(2-phenylethoxy)-6-{[(3,4,5-trihydroxyoxan-2-yl)oxy]methyl}oxane-3,4,5-triol	438.2	5.498	0.03	99.7	0.774	-0.37	0.415
2,3-dinor-8-iso Prostaglandin F2?	348.2	5.98	0.03	91.1	3.067	1.62	0.017
N6,N6,N6-Trimethyl-L-lysine	188.2	0.814	0.03	81.1	0.801	-0.32	0.272
4,5,8-trihydroxy-5-(hydroxymethyl)-3-(3-methoxy-3-oxoprop-1-en-2-yl)-8a-methyl-decahydronaphthalen-2-yl 3,4-dihydroxy-2-methylidenebutanoate	466.2	4.66	0.03	67.5	1.112	0.15	0.134
3-bromo-1??-thiolane-1,1-dione	197.9	0.758	0.03		2.198	1.14	0.030
(2R,3S,4S,5R,6R)-2-({[(2R,3R,4R,5S)-3,4-dihydroxy-5-(hydroxymethyl)oxolan-2-yl]oxy}methyl)-6-{[(2E)-3,7-dimethylocta-2,6-dien-1-yl]oxy}oxane-3,4,5-triol	470.2	6.801	0.03	97.9	1.000	0	0.138
1-Piperideine	83.07	1.302	0.03		0.005	-7.68	0.004
4-Hydroxy-5-methylfuran-3(2H)-one	114	0.955	0.03		0.453	-1.14	0.289
Aniline	93.06	13.76	0.03		0.025	-5.34	0.033
Xanthine	152	1.425	0.03		0.010	-6.61	0.017
L-(-)-methionine	149.1	1.423	0.03		1.10	0.13	0.009
dehydroretinaldehyde	282.2	5.39	0.03		0.058	-4.11	0.014
Cafestol	316.2	9.758	0.03	98.5	6.536	2.71	0.017
Acetylcholine	145.1	1.01	0.03	87.9	0.203	-2.3	0.028
2-Methoxy-1-(3-methyl-2-buten-1-yl)-4-[(Z)-2-phenylvinyl]benzene	278.2	9.646	0.03		0.004	-7.89	0.016
NP-002322	334.2	9.238	0.03	99.4	20.408	4.34	0.000
Cyclopentanone	84.06	6.217	0.03		0.010	-6.58	0.004
Asparagine	132.1	0.881	0.03	94.5	2.01	1.01	0.420
4-Guanidinobutyric acid	145.1	1.229	0.03	89.6	0.903	-0.15	0.446
DL-Carnitine	161.1	1.003	0.03	67.6	0.394	-1.35	0.041
alpha-D-Glucopyranosyl 2-O-(2-methylbutanoyl)-alpha-D-glucopyranoside	448.2	5.073	0.03	89.1	1.036	0.05	0.122

Name	MW (Da)	RT (min)	Area (AU×10^9)	mzCloud	Ratio	Log2FC	Adj. *p*-value
GLIMEPIRIDE, CIS-	490.2	5.541	0.03		1.114	0.16	0.410
2-(Hexopyranosyloxy)-2-methylbutanenitrile	261.1	1.373	0.03		0.022	-5.53	0.001
5-Pentylresorcinol	180.1	8.364	0.03		0.005	-7.77	0.007
5-Methoxy-3-indoleaceate	205.1	5.642	0.03		0.010	-6.71	0.007
DL-Arginine	174.1	0.824	0.03	93.4	0.011	-6.52	0.006
Maltol	126	4.424	0.03	99.5	4.348	2.12	0.021
1-Pentofuranosyl-2,4(1H,3H)-pyrimidinedione	244.1	1.443	0.03		0.012	-6.4	0.025
Catalpol	362.1	3.581	0.03		5.587	2.48	0.017
Kahweol	314.2	9.64	0.03	98.3	3.802	1.93	0.024
1-Methylguanine	165.1	1.412	0.02	98.3	0.660	-0.6	0.247
3-[(2H-1,3-benzodioxol-5-yl)methyl]-4-[(3,4-dimethoxyphenyl)methyl]oxolan-2-one	408.1	2.487	0.02	94.6	0.905	-0.14	0.167
4beta-Phorbol	364.2	5.544	0.02		0.026	-5.26	0.000
(2E)-3-(3,4-Dimethoxyphenyl)acrylic acid	208.1	6.826	0.02		0.012	-6.35	0.010
4-Indolecarbaldehyde	145.1	6.625	0.02		0.966	-0.05	0.762
Scopoletin	192	5.788	0.02	93.3	0.898	-0.15	0.124
Skatole	131.1	4.531	0.02	95.4	0.005	-7.76	0.000
Homo-L-arginine	188.1	0.872	0.02		0.026	-5.27	0.013
3,4-Divanillyltetrahydrofuran	344.2	5.21	0.02		0.014	-6.11	0.003
Capsi-amide	269.3	13.86	0.02		0.011	-6.53	0.014
biotinamide	243.1	0.967	0.02		0.100	-3.32	0.017
RG5927903	210.2	5.763	0.02		0.019	-5.74	0.010
S-methylglutathione	321.1	2.012	0.02		0.823	-0.28	0.063
3-Hydroxy-2-methylpyridine	109.1	1.359	0.02		6.369	2.67	0.005
5,6-dihydroxy-9,12,13-trimethyl-14-(2-methylpropyl)-2H,5H,6H,7H,8H,13H,13aH,14H,15H,16H,16bH-oxacyclododeca[3,2-e]isoindole-2,16-dione	417.3	6.244	0.02	74.4	90.909	6.5	0.000
(3E)-4-methyl-3-(phenylmethylidene)-2,3,4,5-tetrahydro-1H-1,4-benzodiazepine-2,5-dione	300.1	2.977	0.02	93.8	5.076	2.34	0.018
Pyrogallol	126	6.215	0.02		0.014	-6.18	0.019
DPH	389.1	9.745	0.02	60.5	1.13	0.18	0.034
2'-deoxymugineic acid	304.1	0.881	0.02		0.097	-3.37	0.054
gamma-Glutamylvaline	246.1	1.433	0.02		1.12	0.16	0.013
imazamox	305.1	4.939	0.02		0.339	-1.56	0.029
Trichloroacetic acid	161.9	4.728	0.02		0.545	-0.88	0.453
iodoanisole	234	0.766	0.02		0.053	-4.25	0.050
O-heptanoylcarnitine	273.2	5.277	0.02		0.027	-5.19	0.040
Taranabant	515.2	0.951	0.02		1.605	0.68	0.180
7-hydroxy-6-methoxy-2H-chromen-2-one	192	4.824	0.02	72.5	1.067	0.09	0.266
N6-Acetyl-L-lysine	188.1	0.998	0.02	78.6	0.338	-1.56	0.044
g-Guanidinobutyrate	145.1	1.245	0.02		0.053	-4.23	0.023
Botrydial	310.2	9.265	0.02		0.005	-7.54	0.095
(2Z)-5-(1,2,4a,5-tetramethyl-7-oxo-1,2,3,4,4a,7,8,8a-octahydronaphthalen-1-yl)-3-methylpent-2-enoic acid	340.2	6.03	0.02	86	38.462	5.27	0.002
2-Linoleoyl glycerol	336.3	11.26	0.02	90.3	4.082	2.03	0.032
TI0889000	330.2	6.882	0.02		0.019	-5.75	0.017

Name	MW (Da)	RT (min)	Area (AU×10^9)	mzCloud	Ratio	Log2FC	Adj. *p*-value
ϒ-Glutamylcysteine	250.1	1.382	0.02	87.6	1.26	0.33	0.040
6-hydroxy-1H-indole-3-acetamide	190.1	3.804	0.02		0.006	-7.48	0.007
(E)-p-coumaric acid	164	5.139	0.02		0.022	-5.48	0.014
2-Oxo-3-(phosphonooxy)propyl decanoate	324.1	5.139	0.02		1.538	0.62	0.108
1-Aminocyclohexanecarboxylic acid	143.1	2.803	0.02		7.634	2.93	0.007
N-α-L-Acetyl-arginine	216.1	1.251	0.02	97.1	0.907	-0.14	0.112
N-Methyl-1H-indole-3-propanamide	202.1	4.309	0.02		0.081	-3.63	0.062
Pirfenidone	185.1	3.748	0.02		0.009	-6.76	0.017
Pyrrole-2-carboxylic acid	111	1.396	0.02	86.5	20.408	4.36	0.003
A-12(13)-EpODE	294.2	7.717	0.02		0.009	-6.72	0.007
6-Hydroxypicolinic acid	139	1.396	0.02		6.757	2.76	0.006
Zeatin-7-N-glucoside	381.2	1.514	0.02	82.9	1.082	0.11	0.260
D-PANTOTHENIC ACID	219.1	4.158	0.02		0.003	-8.25	0.010
DEET	191.1	8.178	0.02		3.279	1.71	0.051
N-Acetylvaline	159.1	3.173	0.02		0.012	-6.39	0.015
methyl 2,8-dihydroxy-6-(hydroxymethyl)-9-oxo-2,9-dihydro-1H-xanthene-1-carboxylate	340.1	3.567	0.02	84	0.639	-0.65	0.369
(6Z)-1,7-Diphenyl-6-hepten-3-ol	266.2	7.244	0.02		0.007	-7.14	0.007
THTC	132	1.423	0.02		0.009	-6.73	0.003
8-hydroxy-7-methylguanine	181.1	0.745	0.02		0.892	-0.16	0.596
trans-Aconitic acid	174	1.401	0.02	64.4	0.850	-0.23	0.012
NP-014517	352.1	4.113	0.02	99.7	0.684	-0.55	0.788
9-methoxy-7-(4-{[(2S,3R,4S,5S,6R)-3,4,5-trihydroxy-6-(hydroxymethyl)oxan-2-yl]oxy}phenyl)-2H,8H-[1,3]dioxolo[4,5-g]chromen-8-one	496.1	5.805	0.02	61.4	0.962	-0.06	0.240
Minoxidil	209.1	2.301	0.02		0.005	-7.69	0.008
L-(+)-Valine	117.1	1.307	0.02		1.16	0.21	0.006
(3,4,5-trihydroxy-6-{[4-(2,6,6-trimethyl-4-oxocyclohex-2-en-1-yl)butan-2-yl]oxy}oxan-2-yl)methyl 3,4,5-trihydroxybenzoate	546.2	5.208	0.02	98.8	1.199	0.26	0.063
NP-003432	450.2	8.929	0.02	81.4	27.027	4.77	0.002
Cytosine	111	1.003	0.02	97	10.870	3.44	0.000
Tetranor-12(S)-HETE	248.2	11.18	0.02	76.5	0.006	-7.32	0.006
6-Methoxyquinoline	159.1	2.396	0.02		0.700	-0.52	0.208
7-Aminonimetazepam	265.1	5.486	0.02	85.8	15.385	3.94	0.000
Penicillin F	312.1	4.391	0.02		0.076	-3.73	0.016
Phloridzin	436.1	5.541	0.02		0.338	-1.56	0.026
5,10,15-trimethyl-4,9,13-trioxatetracyclo[10.3.0.0Âl,â◎µ.0â◎¸,Âaâ◎°]pentadec-1(15)-en-14-one	246.1	4.654	0.02	90.8	1.107	0.15	0.206
epi-jasmonic acid	210.1	5.228	0.02		0.722	-0.47	0.590
Bayer E 39 Soluble	338.1	2.031	0.02		0.032	-4.97	0.021
3-Methylxanthine	166	3.317	0.02	98.2	0.046	-4.45	0.043
Norharman	168.1	5.181	0.02	99.5	0.851	-0.23	0.183
Indole-3-pyruvic acid	203.1	2.145	0.02	82.8	1.200	0.26	0.077
4-Fluorocyclohexadiene-cis,cis-1,2-diol	130	9.639	0.02		1.825	0.87	0.041
2-[(2R,4aR,8R,8aR)-8-hydroxy-4a,8-dimethyl-decahydronaphthalen-2-yl]prop-2-enoic acid	290.1	5.207	0.02	84.2	1.425	0.51	0.059
Alprenolol	249.2	8.382	0.02		0.954	-0.07	0.131

Name	MW (Da)	RT (min)	Area (AU×10⁹)	mzCloud	Ratio	Log2FC	Adj. *p*-value
(4S,5R,8R,12R)-12-hydroxy-12-(hydroxymethyl)-3,4-dimethyl-11-oxo-10-oxatricyclo[6.4.0.0Âa,â©µ]dodeca-2,6-diene-7-carboxylic acid	332.1	0.871	0.02	73.1	14.925	3.9	0.001
Sulfabenzamide	276.1	6.268	0.02		0.031	-5	0.013
1',4,4''',8''-Tetrahydroxy-2,2''',3',6''-tetramethyl-1,2':7',2'':7'',1'''-quaternaphthalene-1'',4'',5,5',5''',8,8',8'''-octone	746.1	5.016	0.02		0.876	-0.19	0.493
9,16-dihydroxy-6-(hydroxymethyl)-2,6,11-trimethyl-14-oxo-15-oxatetracyclo[8.7.0.0Â?,â©·.0ÂaÂ?,Âaâ©¶]heptadec-12-en-8-yl acetate	390.2	6.43	0.02	64.5	0.800	-0.32	0.145
L(-)-Pipecolinic acid	129.1	0.785	0.02	99.7	0.030	-5.04	0.014
Histamine	111.1	18.12	0.02		0.005	-7.62	0.019
3-(2-Amino-4-pyrimidinyl)alanine	182.1	0.881	0.02		7.407	2.89	0.000
3-[(7,8-Dihydro-3H-purin-6-ylamino)methyl]phenol	243.1	1.376	0.02		16.129	4.02	0.000
Tryptamine	160.1	4.832	0.02	95.3	0.763	-0.39	0.119
Salicylic acid	138	3.633	0.02	87.5	0.014	-6.17	0.082
Dicyclohexylamine	181.2	5.961	0.02		0.051	-4.31	0.019
Safrole	162.1	6.808	0.01		0.083	-3.59	0.079
2-Methylthiazolidine	103	0.879	0.01		1.980	0.99	0.175
3-hydroxy-8-methoxy-3-methyl-1,2,3,4,7,12-hexahydrotetraphene-1,7,12-trione	318.1	1.615	0.01	81.9	0.045	-4.46	0.003
2595	192.2	5.765	0.01		0.029	-5.13	0.008
4-hydroxy-6-[2-(2-methyl-1,2,4a,5,6,7,8,8a-octahydronaphthalen-1-yl)ethyl]oxan-2-one	314.2	4.538	0.01	79.9	0.024	-5.41	0.006
NP-017061	334.2	5.415	0.01	93	0.035	-4.84	0.013
1,4a-Dimethyl-8-methylenegibbane-1,10-dicarboxylate	330.2	5.547	0.01		5.291	2.41	0.036
5,7-dihydroxy-6-methoxy-2-(4-{[(2S,3R,4S,5S,6R)-3,4,5-trihydroxy-6-(hydroxymethyl)oxan-2-yl]oxy}phenyl)-4H-chromen-4-one	462.1	4.506	0.01	91.4	1.558	0.64	0.105
17beta-Methylestra-1,3,5(10)-trien-3-ol	270.2	5.768	0.01		0.024	-5.36	0.015
N-feruloylglycine	251.1	5.641	0.01		0.011	-6.57	0.016
porphobilinogen	226.1	4.124	0.01		0.006	-7.29	0.037
NP-003399	212.1	6.037	0.01	69.1	7.407	2.89	0.003
Rebimastat	499.3	5.667	0.01		4.184	2.07	0.012
N6-Methyladenine	149.1	1.646	0.01	98.6	4.673	2.22	0.012
3-Feruloylquinic acid	368.1	6.712	0.01		0.020	-5.67	0.011
(2R,3S,4S,5R,6S)-2-(hydroxymethyl)-6-[(1R,12R)-5,7,11,19-tetraoxapentacyclo[10.8.0.0Â?,Âaâ©°.0â©´,â©¸.0ÂaÂl,Âaâ©¸]icosa-2(10),3,8,13,15,17-hexaen-16-yloxy]oxane-3,4,5-triol	468.1	3.832	0.01	77	1.992	0.99	0.108
2-{[4-(1,3-Benzodioxol-5-yl)-1-(4-methylphenyl)-1H-imidazol-2-yl]sulfanyl}-N-(3,5-dimethylphenyl)acetamide	471.2	0.959	0.01		1.439	0.52	0.024
2-acetamido-5-oxovalerate	172.1	18.16	0.01		0.989	-0.02	0.999
Mesalamine	153	2.486	0.01		1.425	0.51	0.020
4-Aminobiphenyl	169.1	5.443	0.01		8.000	3	0.011
(1S,4aS,6S,7R,7aS)-6-hydroxy-7-methyl-1-{[(2S,3R,4S,5S,6R)-3,4,5-trihydroxy-6-(hydroxymethyl)oxan-2-yl]oxy}-1H,4aH,5H,6H,7H,7aH-cyclopenta[c]pyran-4-carboxylic acid	398.1	1.657	0.01	96.5	0.884	-0.18	0.233
DIMBOA	211	1.659	0.01		14.706	3.88	0.004
(2S)-2-Piperazinecarboxamide	129.1	1.312	0.01		0.023	-5.42	0.079
2-{[1-hydroxy-1-(4-methoxyphenyl)propan-2-yl]oxy}-6-(hydroxymethyl)oxane-3,4,5-triol	366.1	5.951	0.01	63.9	0.052	-4.26	0.008
(±)17(18)-EpETE	318.2	6.192	0.01	64.4	0.042	-4.57	0.045
NP-020363	304.1	6.724	0.01	62.3	1.101	0.14	0.087
delta-guanidinovaleric acid	159.1	1.563	0.01		0.968	-0.05	0.334

Name	MW (Da)	RT (min)	Area (AU×10^9)	mzCloud	Ratio	Log2FC	Adj. *p*-value
methyl (1S,4aR,7aR)-4a-hydroxy-7-(hydroxymethyl)-1-{[(2S,3R,4S,5S,6R)-3,4,5-trihydroxy-6-(hydroxymethyl)oxan-2-yl]oxy}-1H,4aH,5H,7aH-cyclopenta[c]pyran-4-carboxylate	426.1	3.169	0.01	93.1	0.908	-0.14	0.691
EKK	403.2	6.292	0.01	69.7	0.87	-0.2	0.000
4'-Methoxyacetophenone	150.1	5.466	0.01	90.1	2.451	1.29	0.089
(4E)-4-(2-Oxoethylidene)-1,2,3,4-tetrahydro-2,6-pyridinedicarboxylate	209	1.904	0.01		1.852	0.89	0.326
(4Z)-4-(2-Amino-5-oxo-1,5-dihydro-4H-imidazol-4-ylidene)-4,5,6,7-tetrahydropyrrolo[2,3-c]azepin-8(1H)-one	245.1	5.393	0.01		1.379	0.46	0.083
Methylphenobarbital	246.1	5.949	0.01		0.395	-1.34	0.085
D-(+)-Pipecolinic acid	129.1	0.786	0.01	99.9	0.856	-0.22	0.171
Boc-Asn-Oh	232.1	1.348	0.01		0.016	-5.94	0.012
(E)-4-Methoxycinnamic acid	178.1	6.266	0.01		0.024	-5.37	0.020
NP-018660	208.1	7.203	0.01	74.7	5.495	2.46	0.013
(9R,10R)-10-(acetyloxy)-8,8-dimethyl-2-oxo-2H,8H,9H,10H-pyrano[2,3-h]chromen-9-yl 2-methylbutanoate	426.1	3.064	0.01	99.1	0.047	-4.4	0.024
[(2R,3S,4S,5R,6S)-3,4,5-trihydroxy-6-(4-hydroxyphenoxy)oxan-2-yl]methyl (2E)-3-(3,4-dihydroxyphenyl)prop-2-enoate	456.1	5.781	0.01	71.8	1.546	0.63	0.121
gibberellin A53	348.2	6.188	0.01		0.022	-5.49	0.004
P-DMEA	169.1	9.681	0.01		1.138	0.19	0.154
4-Hydroxybenzoic acid	138	3.657	0.01	94.2	0.883	-0.18	0.154
Stearidonic acid	276.2	7.717	0.01		0.018	-5.77	0.016
NP-003749	420.2	4.131	0.01	69	1.107	0.15	0.171
NP-019410	520.3	6.701	0.01	70.9	1.264	0.34	0.213
3-Pyridinol	95.04	1.462	0.01	93.4	12.195	3.61	0.000
alpha-Ketoglutaric acid	146	1.395	0.01		0.029	-5.08	0.008
Piperazine	86.08	17.72	0.01		0.022	-5.51	0.019
Pregnanetriol	336.3	11.04	0.01		0.218	-2.2	0.086
(11E)-15-Oxo-11-icosenoic acid	324.3	14.22	0.01		1.256	0.33	0.124
(1R,2R,6R,9R)-2,11,11-trimethyl-3-oxotricyclo[4.3.2.0Âa,â◎µ]undecane-9-carboxylic acid	288.1	3.86	0.01	99.6	0.029	-5.08	0.012
NP-012534	306.1	6.413	0.01	98.2	0.535	-0.9	0.377
NP-017624	458.2	6.282	0.01	87.6	5.319	2.41	0.072
NP-008515	286.1	5.871	0.01	62.4	0.123	-3.02	0.011
Benserazide	257.1	1.374	0.01		0.073	-3.77	0.015
Methyldienolone	286.2	6.017	0.01	73.3	1.159	0.21	0.247
(3S,4aR,5R,6R)-3,6-dihydroxy-4a,5-dimethyl-3-(prop-1-en-2-yl)-2,3,4,4a,5,6,7,8-octahydronaphthalen-2-one	272.1	8.675	0.01	88.8	0.837	-0.26	0.251
gamma-Glutamylvaline	246.1	4.326	0.01		0.204	-2.3	0.076
MFCD00060079	234.1	4.126	0.01		0.826	-0.28	0.540
N-Acetyl-L-glutamic acid	189.1	1.642	0.01		0.152	-2.71	0.026
4-methoxy-7-({[(2R,3R,4S,5S,6R)-3,4,5-trihydroxy-6-(hydroxymethyl)oxan-2-yl]oxy}methyl)-5H-furo[3,2-g]chromen-5-one	408.1	2.586	0.01	60.8	6.667	2.74	0.000
Cuminaldehyde	148.1	5.231	0.01	64.7	1.117	0.16	0.124
Z-Leu-OH	265.1	4.446	0.01		0.016	-5.92	0.019
NP-020597	382.2	6.856	0.01	97.9	26.316	4.71	0.000
DL-Lysine	146.1	0.787	0.01		1.16	0.21	0.014
6-hydroxy-4a-(hydroxymethyl)-5-methyl-3-(prop-1-en-2-yl)-2,3,4,4a,5,6,7,8-octahydronaphthalen-2-one	272.1	8.671	0.01	96.9	0.009	-6.79	0.027
MFCD00039529	78.01	18.17	0.01		1.064	0.09	0.157

Name	MW (Da)	RT (min)	Area (AU×10^9)	mzCloud	Ratio	Log2FC	Adj. *p*-value
Cortisone	360.2	6.854	0.01	70.3	10.526	3.4	0.000
Sorbic acid	112.1	8.749	0.01		0.028	-5.13	0.067
Trolox	250.1	9.927	0.01		0.019	-5.73	0.023
1,2,3,4-Tetrahydro-3-isoquinolinecarboxylic acid	177.1	4.002	0.01		5.181	2.38	0.006
YWA1	276.1	6.066	0.01		0.093	-3.43	0.026
1,4,5,6-tetrahydro-6-oxonicotinic acid	141	1.897	0.01		0.017	-5.87	0.014
p-cymene	134.1	5.757	0.01		0.076	-3.72	0.008
2515	252.2	7.868	0.01		0.010	-6.67	0.005
Valylproline	214.1	3.933	0.01	94.4	2.809	1.49	0.013
Citric acid	192	1.408	0.01		0.028	-5.18	0.014
(11E,15Z)-9,10,13-trihydroxyoctadeca-11,15-dienoic acid	350.2	7.376	0.01	98.1	3.906	1.97	0.008
1-(4-Amino-4-carboxybutanoyl)-2-piperidinecarboxylic acid	258.1	1.836	0.01		0.017	-5.87	0.015
(2R,3R,3aR,5R)-5,7-dimethoxy-2-(7-methoxy-2H-1,3-benzodioxol-5-yl)-3-methyl-3a-(prop-2-en-1-yl)-2,3,3a,4,5,6-hexahydro-1-benzofuran-6-one	424.2	5.237	0.01	96.4	0.826	-0.28	0.111
Serotonin	176.1	2.706	0.01	60.8	0.607	-0.72	0.002
[(2R,3S,4S,5R,6S)-3,4,5-trihydroxy-6-[(5-hydroxy-2-methyl-4-oxo-4H-chromen-7-yl)oxy]oxan-2-yl]methyl (4R)-4-(2-hydroxypropan-2-yl)cyclohex-1-ene-1-carboxylate	542.2	7.072	0.01	67.5	1.295	0.37	0.099
Leu-Leu	244.2	5.159	0.01		1.20	0.26	0.007
2878846	314.2	9.643	0.01		0.057	-4.13	0.116
(15E)-15-ethylidene-18-(methoxycarbonyl)-17-methyl-12-oxo-10,17-diazatetracyclo[12.3.1.0Âl,ÂaÂa.0â◎´,â◎a]octadeca-3(11),4,6,8-tetraen-17-ium-17-olate	368.2	9.776	0.01	68.2	15.385	3.94	0.000
NP-004400	578.2	6.057	0.01	94.9	1.395	0.48	0.076
Coenzyme Q2	318.2	9.775	0.01	90.6	4.831	2.27	0.015
NP-019988	176	4.157	0.01	73.2	0.664	-0.59	0.518
Cortisol	362.2	7.328	0.01		0.040	-4.66	0.017
2'-Deoxyadenosine	273.1	1.438	0.01	95.4	1.172	0.23	0.349
Prunin	434.1	5.089	0.01		0.128	-2.97	0.015
Epanolol	369.2	4.339	0.01		0.833	-0.26	0.336
2-hydroxy-6-[2-hydroxy-6-(hydroxymethyl)-4-methoxyphenoxy]-4-methylbenzoic acid	342.1	4.956	0.01	72.7	1.034	0.05	0.204
(4-{[3,4,5-trihydroxy-6-(hydroxymethyl)oxan-2-yl]oxy}phenyl)methyl 2,3-dihydroxy-2-[(4-hydroxyphenyl)methyl]butanoate	516.2	4.024	0.01	96.2	3.012	1.59	0.113
2-Aminoindan-2-carboxylic acid	177.1	1.508	0.01		6.667	2.74	0.015
procainamide	235.2	2.581	0.01		0.025	-5.32	0.017
Butyrophenone	148.1	9.673	0.01		0.051	-4.29	0.107
NP-007970	556.3	5.765	0.01	96	1.325	0.4	0.190
L-gamma-Glutamyl-L-leucine	260.1	2.65	0.01		1.20	0.26	0.024
NP-007425	492.2	5.586	0.01	81.1	1.642	0.72	0.116
NP-006111	304.1	6.323	0.01	71.1	0.564	-0.83	0.859
Edaravone	174.1	2.746	0.01		0.009	-6.79	0.009
KKK	402.3	5.013	0.01	67.9	0.70	-0.51	0.015
6-(3-hydroxybutan-2-yl)-5-(hydroxymethyl)-4-methoxy-2H-pyran-2-one	266.1	1.627	0.01	62.4	2.058	1.04	0.080
promolate	293.2	5.689	0.01		0.051	-4.3	0.002
pymetrozine	217.1	5.395	0.01		1.046	0.07	0.092
Hexylresorcinol	194.1	7.718	0.01		0.043	-4.55	0.017

Name	MW (Da)	RT (min)	Area (AU×10^9)	mzCloud	Ratio	Log2FC	Adj. *p*-value
2-Guanidinobenzimidazole	175.1	1.319	0.01		17.544	4.13	0.002
laurilsulfate	266.2	5.84	0.01		0.779	-0.36	0.382
D-(−)-Quinic acid	192.1	0.959	0.01		0.330	-1.6	0.009
Bicyclo prostaglandin E2	334.2	6.074	0.01	76.6	0.306	-1.71	0.123
1-{2-[4-(2-Phenyl-6,7,8,9-tetrahydro-5H-cyclohepta[4,5]benzo[1,2-b]furan-3-yl)phenoxy]ethyl}pyrrolidine	451.3	5.694	0.01		11.111	3.47	0.001
NP-020214	310.2	7.869	0.01	84.3	2.545	1.35	0.053
(9E)-5-hydroxy-17-[(1H-indol-3-yl)methyl]-4-methoxy-7,14,15-trimethyl-13-oxa-18-azatetracyclo[9.8.0.0Âa,Âaâ◎¶.0ÂaÂ?,Âaâ◎´]nonadec-9-ene-2,19-dione	528.3	5.59	0.01	81.2	1.010	0.01	0.147
Precocene II	220.1	9.743	0.01		0.058	-4.11	0.241
(2R,3S,4S,5R,6R)-2-({[(2S,3R,4R)-3,4-dihydroxy-4-(hydroxymethyl)oxolan-2-yl]oxy}methyl)-6-(2-phenylethoxy)oxane-3,4,5-triol	454.1	5.496	0.01	91.7	0.718	-0.48	0.288
(9cis)-Retinal	284.2	5.23	0.01	74.5	1.565	0.65	0.112
NP-016582	305.3	11.48	0.01	88	1.590	0.67	0.310
Hexadecanamide	255.3	13.72	0.01	98.3	0.023	-5.42	0.010
Cinnamic acid	148.1	3.893	0.01		0.880	-0.18	0.862
NP-002999	578.2	6.056	0.01	67.6	0.035	-4.83	0.005
Picrocrocin	330.2	8.736	0.01		0.020	-5.68	0.017
O-Acetylserine	147.1	1.746	0.01	83.8	0.871	-0.2	0.167
(1Z)-2-[(Z)-(2-Amino-1-hydroxyethylidene)amino]-4-methyl-N-(1-phosphonovinyl)pentanimidic acid	293.1	1.379	0.01		4.785	2.26	0.001
Hydroxy-1,4-benzoquinone	124	1.974	0.01		0.017	-5.88	0.007
(6-{[3,4-dihydroxy-2,5-bis(hydroxymethyl)oxolan-2-yl]oxy}-3,4,5-trihydroxyoxan-2-yl)methyl (2E)-3-(4-hydroxy-3-methoxyphenyl)prop-2-enoate	540.2	5.07	0.01	83.7	3.559	1.83	0.004
4-Aminophenol	109.1	1.37	0.01		0.064	-3.97	0.065
Andrographolide	332.2	6.846	0.01	81.2	16.949	4.09	0.002
Peldesine	241.1	1.345	0.01		4.167	2.06	0.001
ionene	174.1	5.763	0.01		0.060	-4.06	0.010
Cyclohexene, 6-(2-butenylidene)-1,5,5-trimethyl-, (E,Z)-	176.2	5.442	0.01		0.998	0	0.651
L-Glutathione (reduced)	307.1	1.343	0.01	97.4	1.14	0.19	0.019
NP-003058	420.2	4.129	0.01	76.9	0.014	-6.11	0.017
Nedocromil	371.1	5.879	0.01		0.176	-2.51	0.029
Isovanillic acid	168	4.113	0.01	75	0.837	-0.26	0.067
MFCD01095901	290.2	6.314	0.01		1.161	0.22	0.119
4-oxododecanedioic acid	266.1	4.569	0.01	98	0.436	-1.2	0.696
Aceglutamide	188.1	1.275	0.01		1.022	0.03	0.246
(2R,3R,4S,5S,6R)-2-{[4-(4-hydroxy-2,6,6-trimethylcyclohex-1-en-1-yl)butan-2-yl]oxy}-6-(hydroxymethyl)oxane-3,4,5-triol	396.2	6.991	0.01	84.4	1.316	0.4	0.145
(2E)-5-methyl-2-phenylhex-2-enal	188.1	5.233	0.01		0.026	-5.29	0.016
Tetranor-12R-HETE	248.2	8.721	0.01	62	2.519	1.33	0.056
trifloxystrobin	408.1	2.474	0.01		3.497	1.81	0.007
Succinyladenosine	383.1	4.093	0.01		0.027	-5.21	0.021
(E)-isoeugenyl benzyl ether	254.1	5.538	0.01		0.487	-1.04	0.036
12-Oxo phytodienoic acid	292.2	7.372	0.01	84.1	4.425	2.14	0.014
N-Acetyl vitamin K5	215.1	4.274	0.01		0.243	-2.04	0.048
Bleekerine	408.2	6.215	0.01		1.385	0.47	0.148
2'-Deoxycytosine	97.06	18.12	0.01		0.014	-6.12	0.017

Name	MW (Da)	RT (min)	Area (AU×10^9)	mzCloud	Ratio	Log2FC	Adj. *p*-value
N-Benzylformamide	135.1	4.598	0.01	81.1	0.182	-2.46	0.023
6-(cyclohexylmethylidene)-6,7,8,9-tetrahydro-5H-benzo[a]cyclohepten-5-one	254.2	5.54	0.01		1.183	0.24	0.446
felbamate	238.1	2.328	0.01		0.032	-4.95	0.000
3-[2-(Hydroxymethyl)-4-methoxyphenyl]-6-methoxy-4-oxo-3,4-dihydro-1(2H)-quinazolinecarbaldehyde	342.1	4.167	0.01		1.041	0.06	0.190
Fleroxacin	369.1	6.624	0.01		0.181	-2.47	0.121
Benzyl butyrate	178.1	9.056	0.01		0.023	-5.43	0.013
Vanillin	152	5.642	0.01	83.1	0.503	-0.99	0.000
NP-016035	456.2	6.307	0.01	61	0.101	-3.3	0.057
(+/-)5(6)-EET Ethanolamide	385.3	6.152	0.01	74.7	5.181	2.38	0.005
L-Histidine	155.1	0.8	0.01	98.3	1.17	0.22	0.012
NP-012381	496.1	6.574	0.01	61.4	0.015	-6.06	0.014
Camptothecin	348.1	6.624	0.01		0.741	-0.43	0.122
(2E)-3-{[(4R,6aR,9S,9aR,9bR)-9-methyl-3,6-dimethylidene-2,8-dioxo-dodecahydroazuleno[4,5-b]furan-4-yl]oxy}-2-(2-hydroxyethylidene)-3-oxopropyl (2E)-4-hydroxy-2-methylbut-2-enoate	496.2	6.148	0.01	95	1.318	0.4	0.295
4-Methoxyaniline	123.1	2.013	0.01		0.046	-4.44	0.014
NP-019547	332.2	9.267	0.01	98.7	0.018	-5.8	0.011
H-Gln(Ph)-OH	222.1	4.576	0.01		0.016	-5.98	0.005
Toluene	92.06	3.902	0.01		0.903	-0.15	0.148
L-Phenylalanine	165.1	5.835	0.01	85.3	0.79	-0.33	0.001
Tetralin	132.1	5.761	0.01		0.072	-3.8	0.007
Adefovir	273.1	5.634	0.01		0.845	-0.24	0.550
gibberellin A12	332.2	5.286	0.01		0.323	-1.63	0.028
Methionine sulfoxide	165	1.295	0.01	72.2	18.182	4.19	0.002
4-oxo-5-phenylpentanoic acid	192.1	5.639	0.01	85.7	0.577	-0.79	0.523
2,6-di-tert-butyl-4-ethylphenol	234.2	7.867	0.01		0.018	-5.79	0.012
FLUMEZAPINE	330.1	5.568	0.01		1.082	0.11	0.127
S-(2-Aminoethyl)cysteine	164.1	18.16	0.01		0.992	-0.01	0.385
Adenosine 3'5'-cyclic monophosphate	329.1	1.561	0.01	69.6	13.699	3.77	0.000
N-Acetylhistamine	153.1	1.485	0.01	72.7	26.316	4.7	0.000
L-(-)-Serine	105	0.88	0.01		1.24	0.31	0.068
(2S,5Z)-2-Amino-5-{[(1S,4Z)-1-carboxy-4-{[(1S)-1-carboxy-2-(methylsulfanyl)ethyl]imino}-4-hydroxybutyl]imino}-5-hydroxypentanoic acid (non-preferred name)	393.1	4.069	0.01		0.329	-1.6	0.072
(1aR,1bR,2R,3R,7R,7aS)-1b,2-dimethyl-7a-(prop-1-en-2-yl)-1aH,1bH,2H,3H,4H,5H,7H,7aH-naphtho[1,2-b]oxirene-3,7-diol	232.1	8.676	0.01	84.1	0.854	-0.23	0.304
1-(3-ethyl-2,4-dihydroxy-6-methoxyphenyl)butan-1-one	238.1	9.746	0.01	67.1	0.027	-5.21	0.020
S-Adenosylhomocysteine	384.1	1.611	0.01	94.5	0.034	-4.88	0.022
2,5-Dipropyl-4-methylthiazole	183.1	6.876	0.01		0.041	-4.6	0.018
PANTOTHENOYLCYSTEINE	322.1	4.043	0.01		0.381	-1.39	0.133
Caprolactam	113.1	4.651	0.01		0.078	-3.67	0.009
Levosimendan	280.1	4.639	0.01		0.060	-4.06	0.019
bendiocarb	223.1	4.594	0.01		0.347	-1.53	0.024
3420	190.1	12.06	0.01		0.041	-4.59	0.014
NP-012387	568.2	4.552	0.01	83.1	0.723	-0.47	0.141

Name	MW (Da)	RT (min)	Area (AU×10^9)	mzCloud	Ratio	Log2FC	Adj. *p*-value
Methyl alpha-aspartylphenylalaninate	294.1	4.089	0.01		0.035	-4.86	0.015
4,4'-Dinitrobibenzyl	272.1	5.815	0.01		0.109	-3.19	0.013
4-decyl-3-hydroxy-5-oxooxolane-2,3-dicarboxylic acid	352.2	8.716	0.01	98.3	0.125	-3	0.028
6-Acetamido-2-oxohexanoic acid	187.1	1.468	0.01		0.104	-3.27	0.010
Stiripentol	234.1	9.543	0.01		0.081	-3.63	0.019
meprobamate	218.1	4.151	0.01		0.012	-6.42	0.012
Androstanolone	290.2	6.314	0.01		0.042	-4.59	0.014
glutethimide	217.1	4.466	0.01		0.023	-5.45	0.022
Indole-3-acetic acid	175.1	2.699	0.01	82.4	0.551	-0.86	0.966
(3aS,5aS,9bR)-5a,9-dimethyl-3-methylidene-2H,3H,3aH,4H,5H,5aH,6H,7H,8H,9bH-naphtho[1,2-b]furan-2,5-dione	228.1	6.348	0.01	86.3	1.748	0.81	0.119
Salsolinol	179.1	3.302	0.01	85.3	25.641	4.68	0.000
Ambrette musk	268.1	1.486	0.01		0.105	-3.25	0.060
Melilotoside	326.1	6.363	0.01		0.037	-4.76	0.006
Gramine	129.1	4.676	0.01	98.9	1.250	0.32	0.087
oxazolidinone	87.03	0.885	0.01		0.286	-1.81	0.014
5-(1,2,4a,5-tetramethyl-7-oxo-1,2,3,4,4a,7,8,8a-octahydronaphthalen-1-yl)-3-methylpentanoic acid	342.2	6.104	0.01	97.1	6.452	2.69	0.008
EUPATORIOCHROMENE	218.1	6.433	0.01		0.077	-3.7	0.088
AMPA	186.1	1.409	0.01		0.063	-4	0.015
(3-Hydroxy-2-oxo-2,3-dihydro-1H-indol-3-yl)acetonitrile	188.1	3.545	0.01		0.011	-6.45	0.014
Dinoseb	240.1	1.939	0.01		0.166	-2.59	0.038
4-Methyl-5-thiazoleethanol	143	6.103	0.01	68.7	1.172	0.23	0.154
NP-013210	206.1	6.945	0.01	65	1.548	0.63	0.168
DC2810000	168.1	6.218	0.01		0.043	-4.53	0.014
Arachidonic acid	304.2	7.284	0.01	78.7	5.405	2.43	0.005
NP-021047	558.3	5.437	0.01	95.1	0.976	-0.04	0.735
4-Vinylcyclohexene	108.1	5.762	0.01		0.090	-3.47	0.007
MFCD12546417	190	2.155	0.01		0.054	-4.21	0.016
1-Naphthol	144.1	9.643	0.01	91.7	2.165	1.11	0.028
(+/-)-Camphor	152.1	5.228	0.01		0.070	-3.84	0.007
5,6,7-Trimethoxy-2H-chromen-2-one	236.1	3.986	0.01		0.019	-5.71	0.005
NP-022454	208.1	6.629	0.01	82.6	0.430	-1.22	0.858
octadec-9-ynoic acid	262.2	13.38	0.01	94.6	3.003	1.58	0.019
NP-006274	274	4.601	0.01	85.8	0.954	-0.07	0.095
Mesalazine	153	2.415	0.01		0.018	-5.79	0.006
WQH	469.2	5.723	0.01	61.7	1.61	0.69	0.019
(1R,2R,4S,16R,17R,20S)-2-hydroxy-13,20-dimethoxy-4,7,17,22,22-pentamethyl-5,10,21,23-tetraoxahexacyclo[18.2.1.0Âa,Âaâ◎·.0â◎´,Âaâ◎¶.0â◎¶,Âaâ◎´.0â◎¸,ÂaÂ?]tricosa-6,8(12),13-trien-11-one	496.2	4.489	0.01	72.1	1.328	0.41	0.063
(9R,10R)-10-hydroxy-8,8-dimethyl-9-{[(2S,3R,4S,5S,6R)-3,4,5-trihydroxy-6-(hydroxymethyl)oxan-2-yl]oxy}-2H,8H,9H,10H-pyrano[2,3-h]chromen-2-one	446.1	6.359	0.01	92.6	1.637	0.71	0.085
Benzoxazole	119	4.469	0.01		0.047	-4.41	0.008
2-(Phosphonatooxy)acrylate	165	0.855	0.01		0.055	-4.17	0.021
3,4-Dihydroxyphenylpropionic acid	199.1	7.28	0.01	76.3	5.208	2.38	0.012

Name	MW (Da)	RT (min)	Area (AU×10^9)	mzCloud	Ratio	Log2FC	Adj. *p*-value
epothilone C	477.3	7.648	0.00		11.494	3.53	0.000
NP-010028	334.2	13.98	0.00	89	1.667	0.74	0.307
NP-021207	272.1	9.927	0.00	77	0.304	-1.72	0.070
(2S)-2-Amino-4-[[(2R)-2-amino-2-carboxyethyl]sulfinyl]butanoic acid	238.1	0.822	0.00		0.127	-2.97	0.011
NP-018536	232.1	8.666	0.00	84.1	0.016	-5.94	0.007
Eriodictyol	288.1	6.207	0.00		0.615	-0.7	0.076
2,3,4,5-tetrahydrodipicolinic acid	171.1	1.648	0.00		0.356	-1.49	0.046
D,L-Camphor	152.1	7.354	0.00	74.2	1.626	0.7	0.076
Karwinaphthol B	288.1	6.719	0.00		0.443	-1.17	0.702
Diphenylamine	169.1	5.468	0.00		0.160	-2.64	0.020
Caffeic acid 3-glucoside	342.1	4.622	0.00		0.043	-4.53	0.025
Epirizole	234.1	1.815	0.00		0.072	-3.8	0.020
2-Hydroxy-2-(2-methylenecyclopropyl)succinic acid	186.1	2.391	0.00		0.093	-3.42	0.075
2'-O-Methyladenosine	281.1	3.717	0.00	65.1	19.608	4.29	0.014
Hypoxanthin	136	2.265	0.00		1.15	0.202	0.009
Diethyl phthalate	222.1	4.527	0.00		0.151	-2.73	0.009
NP-019159	500.2	8.225	0.00	77.1	12.987	3.7	0.001
2,6-Diamino-4-oxo-5-formamidopyrimidine	169.1	1.669	0.00		1.259	0.33	0.121
(−)-nabilone	372.3	6.681	0.00		1.783	0.83	0.006
4-hydroxy-3-[(2E,6E)-4-hydroxy-3,7,11-trimethyldodeca-2,6,10-trien-1-yl]benzoic acid	380.2	7.286	0.00	95.4	14.706	3.89	0.000
Coniferyl ferulate	356.1	6.994	0.00		0.257	-1.96	0.078
NP-015484	474.2	4.585	0.00	89.4	1.064	0.09	0.482
N2-Dimethylguanosine	311.1	3.624	0.00		0.023	-5.43	0.018
NP-007714	515.2	6.032	0.00	75.3	0.931	-0.1	0.163
NP-021084	300.2	8.121	0.00	85.9	1.698	0.76	0.061
LW8000000	184	2.209	0.00		7.407	2.89	0.003
(1S,2S,3R,4S,7R,10S,12R,15S)-4,12-bis(acetyloxy)-1,9,15-trihydroxy-10,14,17,17-tetramethyl-11-oxo-6-oxatetracyclo[11.3.1.0Âl,Âaâ©°.0â©´,â©·]heptadec-13-en-2-yl benzoate	608.2	7.17	0.00	80.3	0.491	-1.02	0.163
NP-021018	248.1	7.077	0.00	85.4	2.183	1.13	0.045
2486876	374.2	6.461	0.00		0.062	-4.01	0.013
tranexamic acid	157.1	1.314	0.00		0.056	-4.15	0.007
N-Acetylcystathionine	264.1	2.076	0.00		0.121	-3.04	0.032
(2R,3S,4S,5R,6R)-2-({[(2S,3R,4R)-3,4-dihydroxy-4-(hydroxymethyl)oxolan-2-yl]oxy}methyl)-6-{[4-(4-hydroxyphenyl)butan-2-yl]oxy}oxane-3,4,5-triol	482.2	5.45	0.00	97.9	1.203	0.27	0.869
5-[4-(3-hydroxy-4-methoxyphenyl)-hexahydrofuro[3,4-c]furan-1-yl]-2-methoxyphenol	340.1	6.989	0.00	83.2	7.937	2.99	0.011
2-Dodecyltetrahydrothiophene	256.2	7.007	0.00		3.003	1.58	0.124
triafungin	222.1	7.986	0.00		14.493	3.86	0.000
(7E)-3,8-Dimethyl-7-decen-1-yl trihydrogen diphosphate	344.1	4.539	0.00		1.376	0.46	0.470
Piceatannol	244.1	8.933	0.00	71	1.416	0.5	0.121
Alanyltyrosine	252.1	3.999	0.00	69.7	0.850	-0.24	0.128
(2S)-3-(4-Hydroxyphenyl)-2-({[(3S,4S,5R)-2,3,4-trihydroxy-5-(hydroxymethyl)tetrahydro-2-furanyl]methyl}amino)propanoic acid (non-preferred name)	343.1	3.619	0.00		0.040	-4.65	0.007
4-METHYL MEIQX	227.1	6.165	0.00		1.812	0.86	0.033

Name	MW (Da)	RT (min)	Area (AU×10^9)	mzCloud	Ratio	Log2FC	Adj. *p*-value
Paradol	278.2	9.769	0.00		0.320	-1.64	0.177
NP-005519	288.1	4.566	0.00	65.2	0.735	-0.44	0.172
(4S)-4-[(6-Carboxyhexanoyl)oxy]-4-(trimethylammonio)butanoate	303.2	8.748	0.00		0.139	-2.85	0.049
(3aR,7aS,8S,9aR)-5,8-dimethyl-3-methylidene-2H,3H,3aH,4H,6H,7H,7aH,8H,9H,9aH-azuleno[6,5-b]furan-2,6-dione	228.1	4.655	0.00	72.2	1.092	0.13	0.120
NP-007065	176	3.979	0.00	71.4	0.092	-3.44	0.003
1-[(4-Amino-2-methyl-5-pyrimidinyl)methyl]pyridinium	201.1	4.19	0.00		30.303	4.92	0.000
