# Supplementary material for: Potential of Arabica Coffee Beans from Northern Thailand: Exploring Antidiabetic Metabolites through Liquid Chromatography with Tandem Mass Spectrometry (LC-MS/MS) Metabolomic Profiling across Diverse Postharvest Processing Techniques
Source: Foods. 2023 Oct 24;12(21):3893. doi: 10.3390/foods12213893 (PMC10648821; doi:10.3390/foods12213893)

Supplementary data S1

1. MS and MS2 chromatogram of untargeted metabolomics analysis

1.1 MS Chromatogram of natural and washed processed coffee beans.

Experimental condition	Biological replication	Technical replication	Intensity (AU)	Chromatogram
Natural process coffee beans	1	1	$1e^{10}$	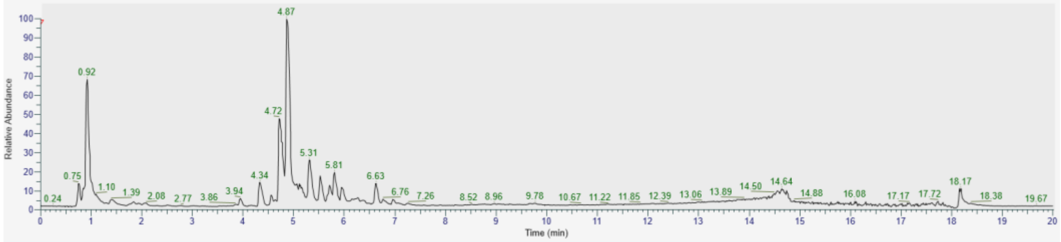
		2	$1e^{10}$	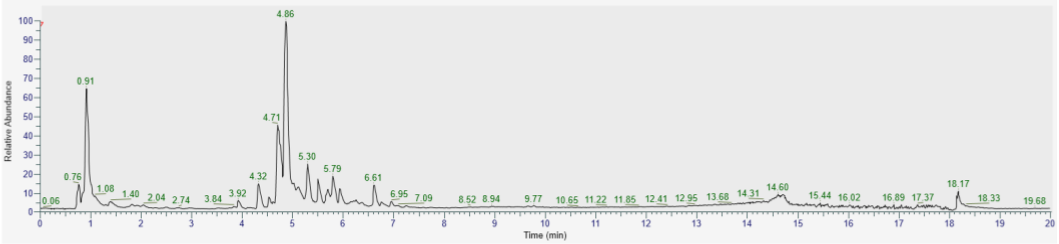
		3	$1e^{10}$	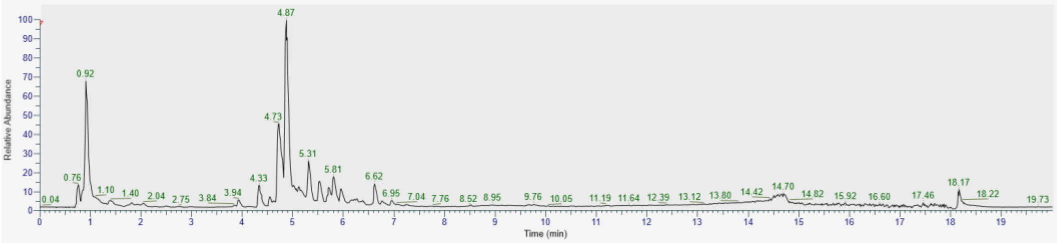
	2	1	$9.1e^{9}$	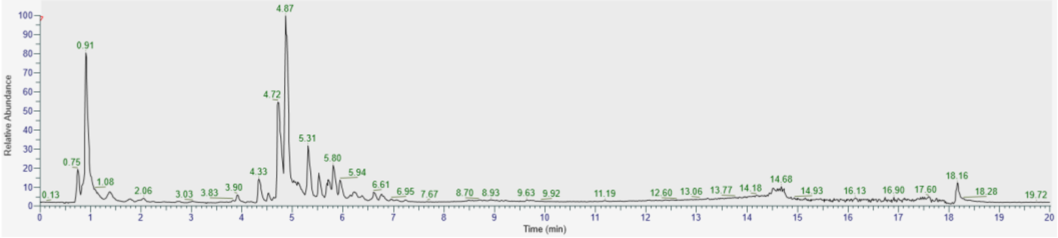

Experimental condition	Biological replication	Technical replication	Intensity (AU)	Chromatogram
		2	$9.6e^9$	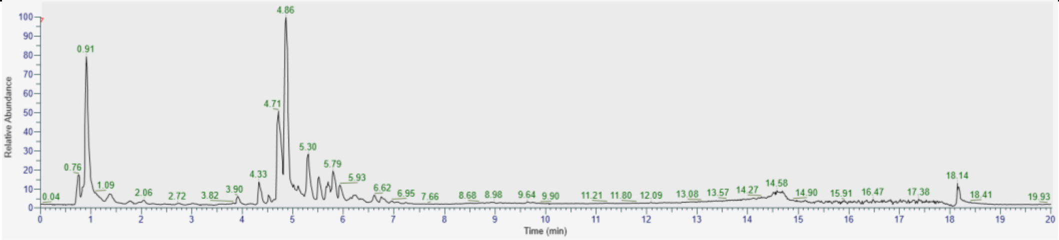
		3	$9.4e^9$	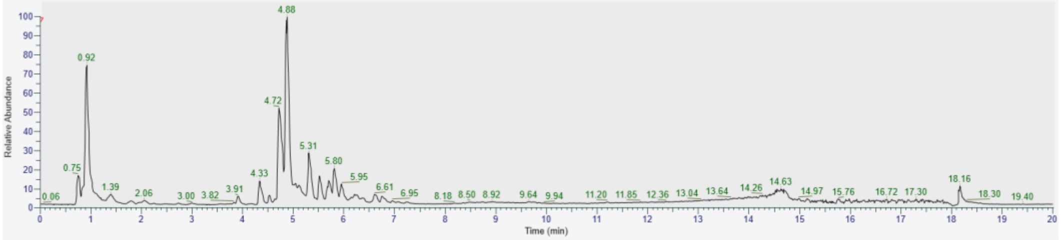
	3	1	$8.5e^9$	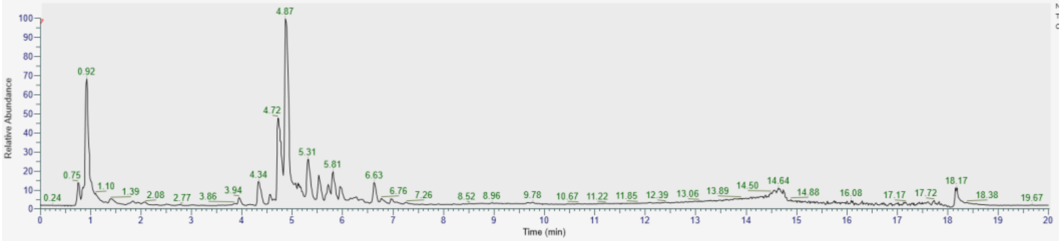
		2	$8.4e^9$	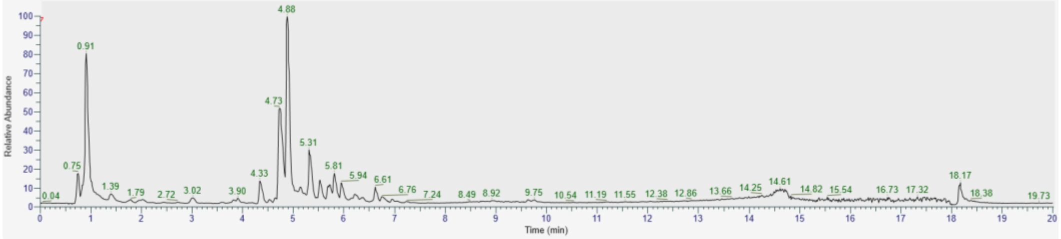
		3	$8.8e^9$	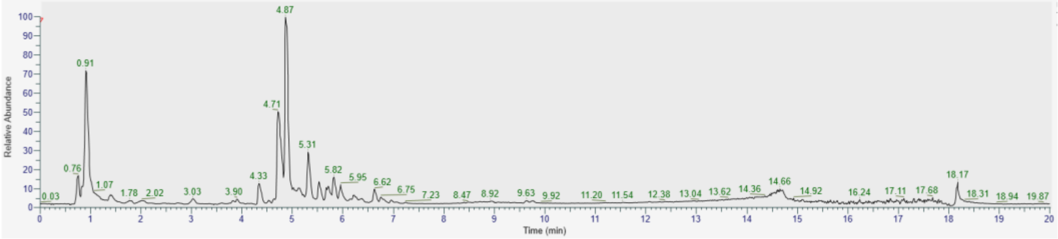

Experimental condition	Biological replication	Technical replication	Intensity (AU)	Chromatogram
Washed process coffee beans	1	1	$9.3e^{9}$	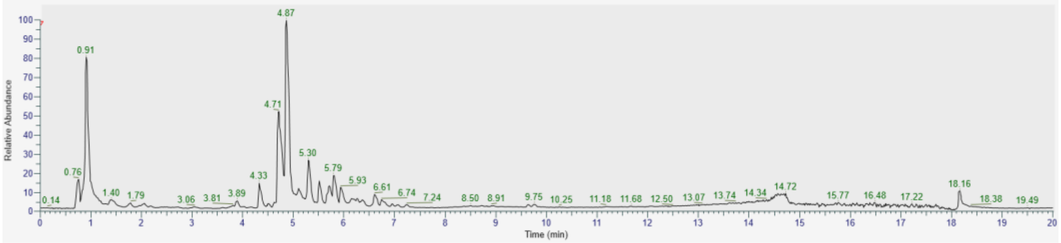
		2	$8.7e^{9}$	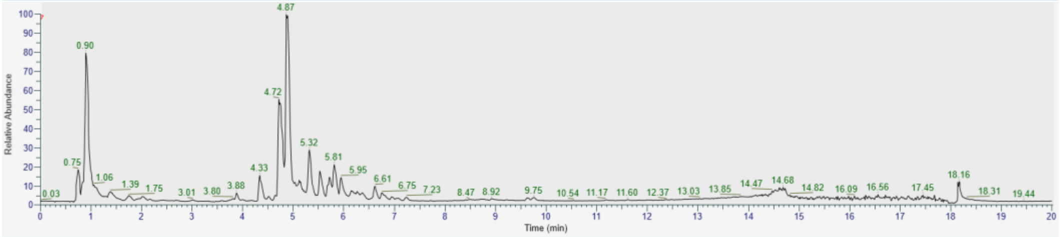
		3	$9.1e^{9}$	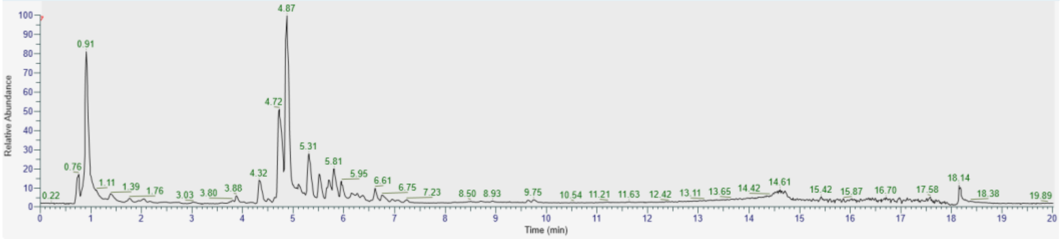
	2	1	$1e^{10}$	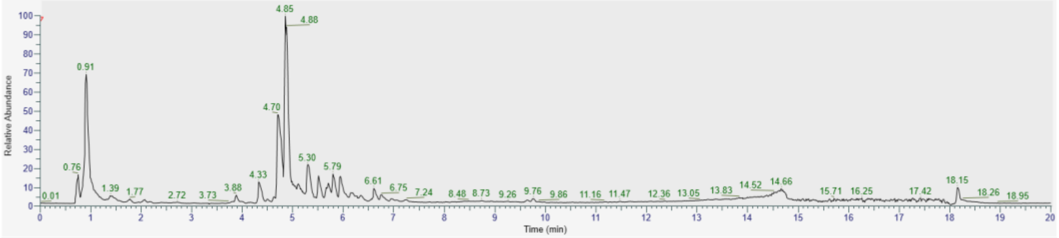
		2	$1e^{10}$	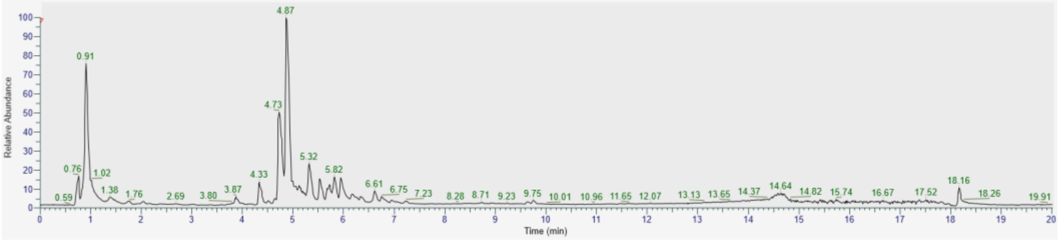

Experimental condition	Biological replication	Technical replication	Intensity (AU)	Chromatogram
		3	$9.7e^9$	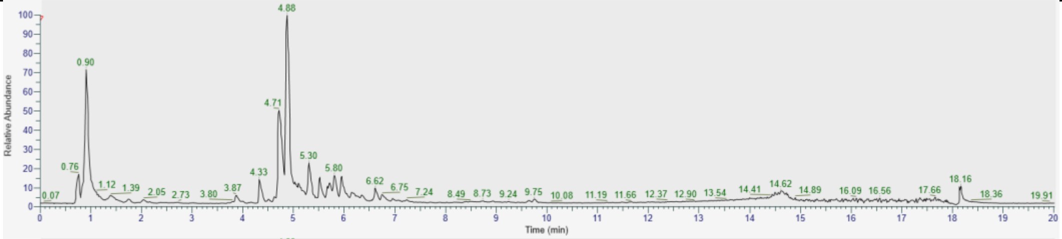
	3	1	$8.5e^9$	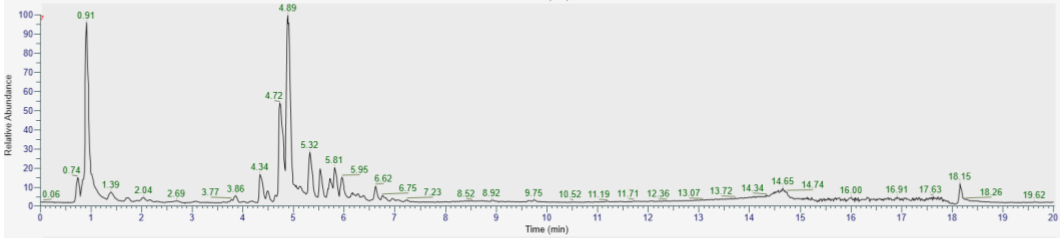
		2	$9.2e^9$	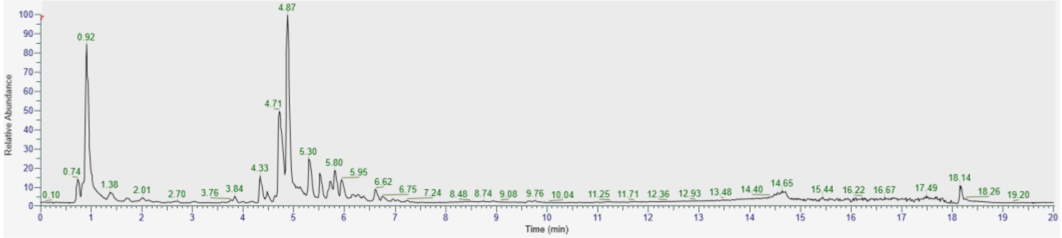
		3	$9e^9$	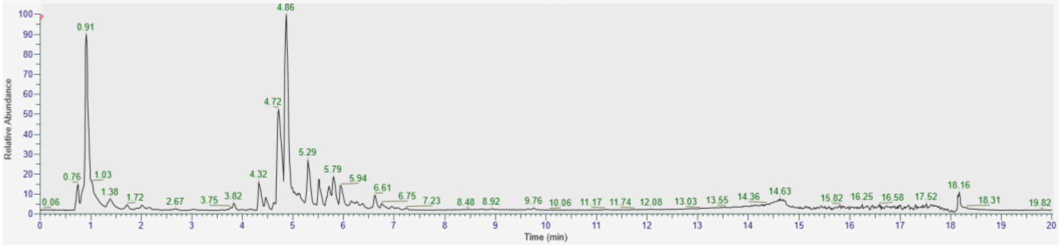

1.2 MS2 Chromatogram of natural and washed processed coffee beans

Experimental condition	Biological replication	Technical replication	Intensity (AU)	Chromatogram
Natural process coffee beans	1	1	$4.6e^{9}$	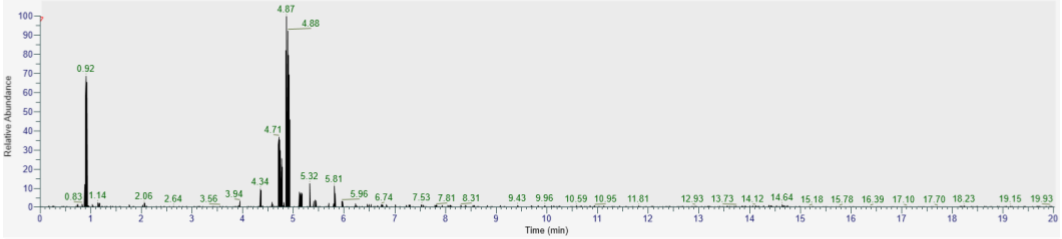
		2	$4.5e^{9}$	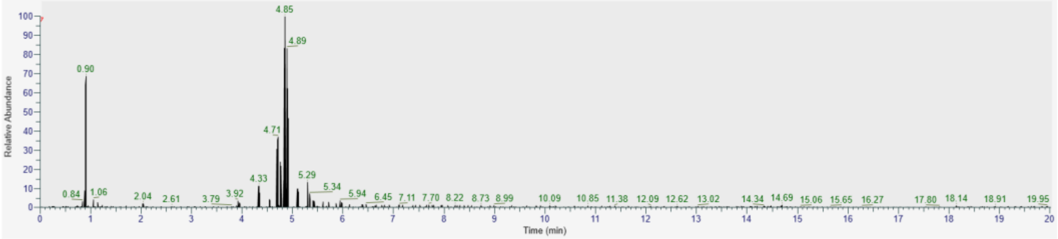
		3	$4.7e^{9}$	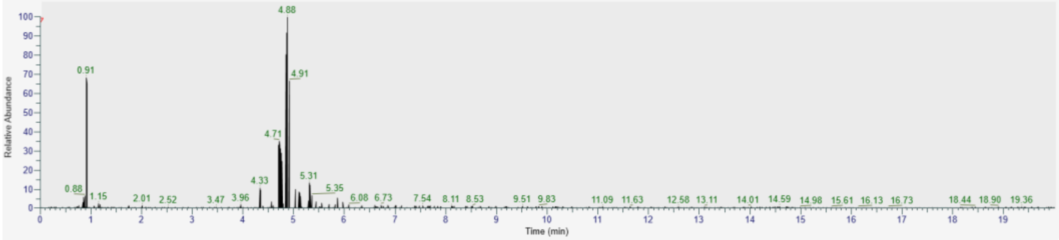
	2	1	$4.2e^{9}$	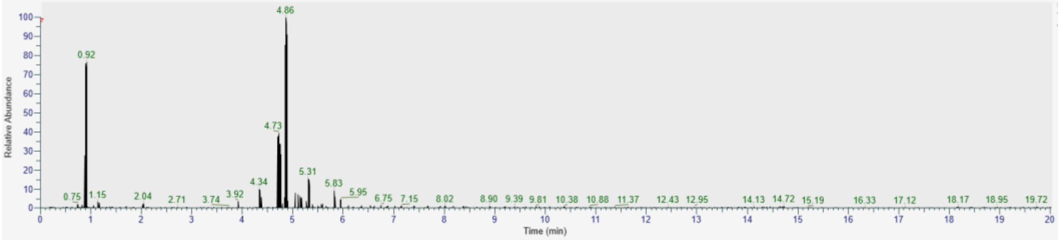
		2	$4e^{9}$	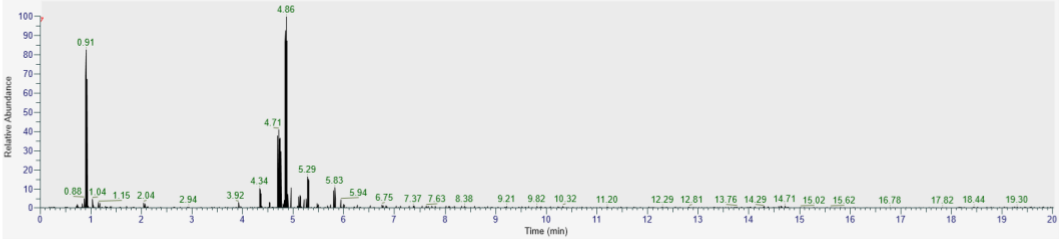

Experimental condition	Biological replication	Technical replication	Intensity (AU)	Chromatogram
		3	$3.8e^9$	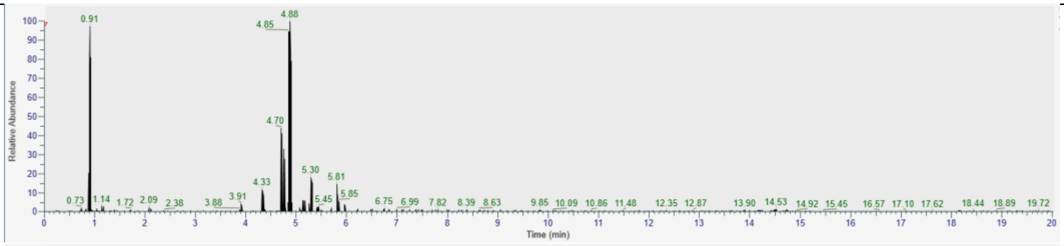
	3	1	$4.6e^9$	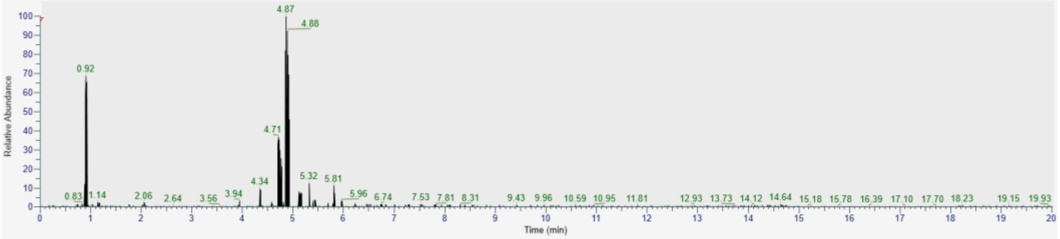
		2	$3.8e^9$	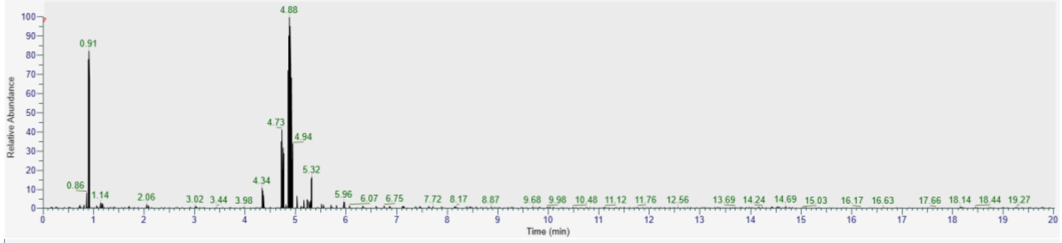
		3	$3.8e^9$	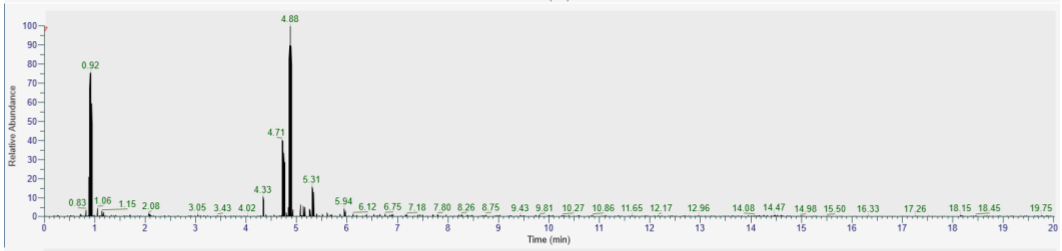
Washed process coffee beans	1	1	$3.8e^9$	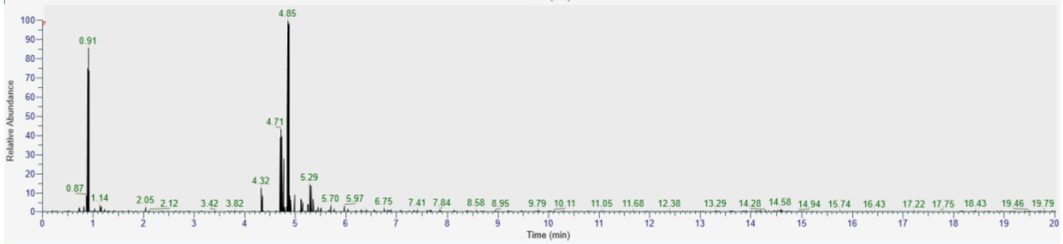

Experimental condition	Biological replication	Technical replication	Intensity (AU)	Chromatogram
		2	$3.8e^9$	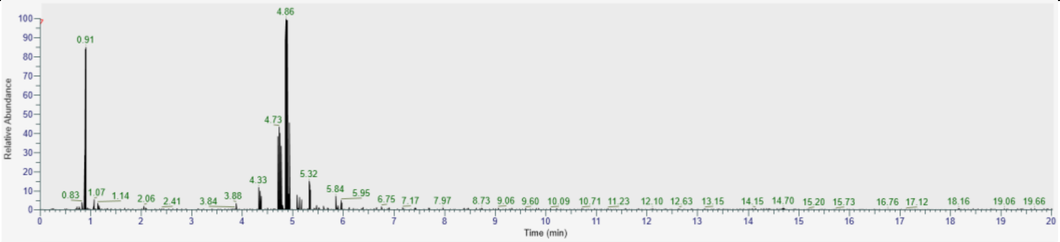
		3	$4.1e^9$	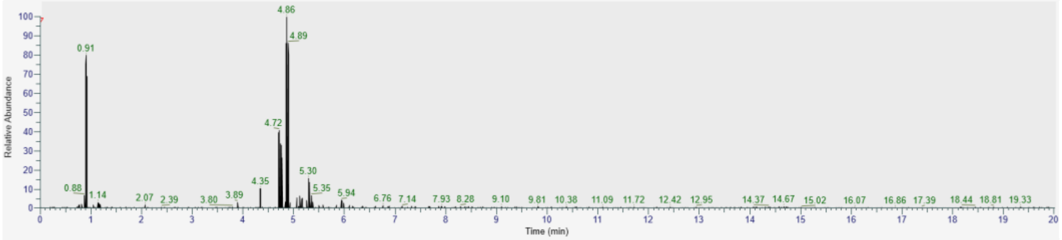
		1	$4.1e^9$	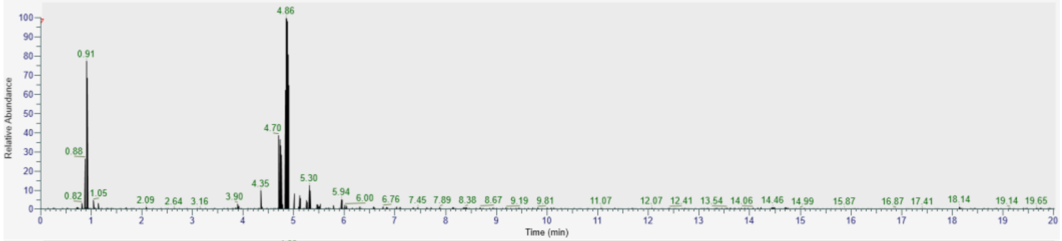
	2	2	$4.3e^9$	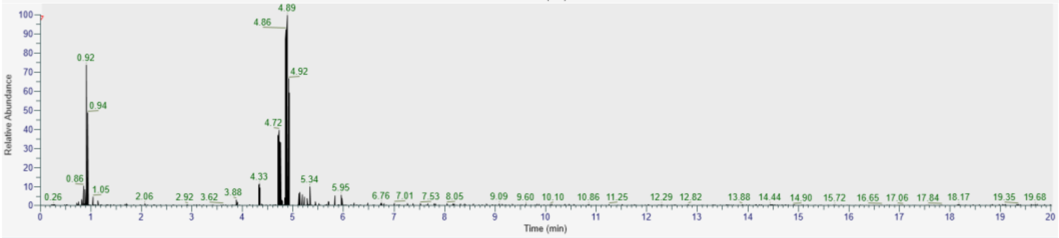
		3	$4.1e^9$	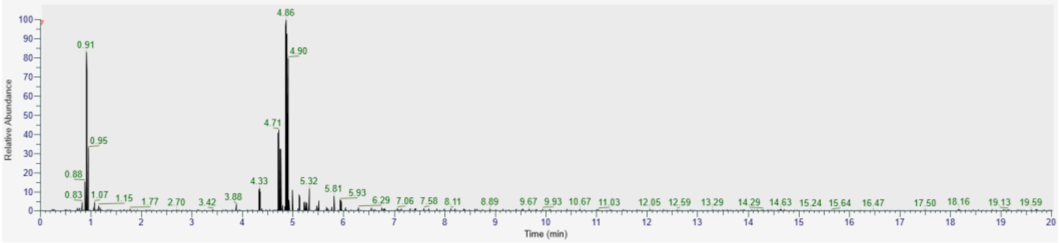

Experimental condition	Biological replication	Technical replication	Intensity (AU)	Chromatogram
	3	1	$3.8e^9$	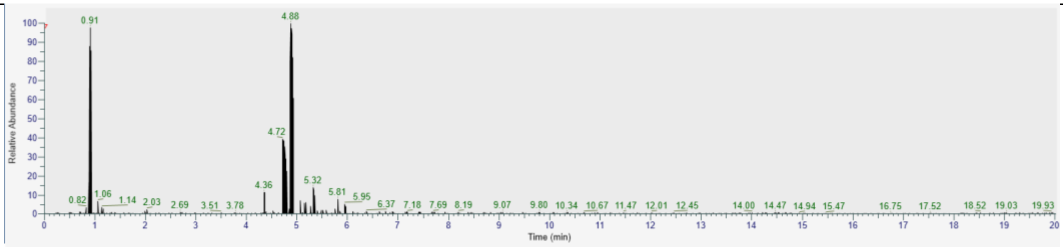
		2	$4.2e^9$	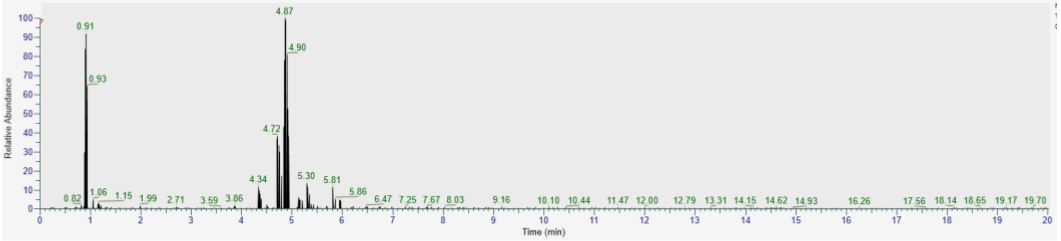
		3	$3.9e^9$	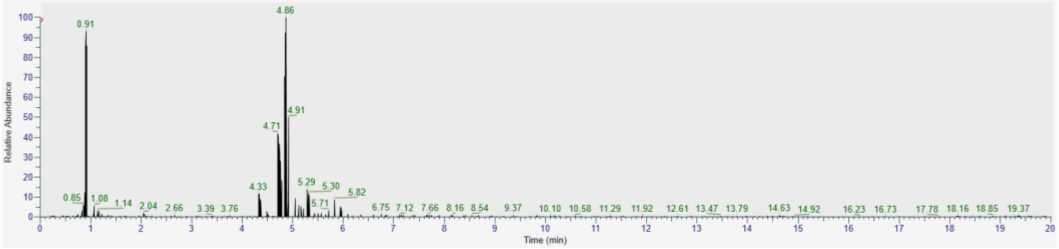

2. Chromatogram of CGA

3.1 MS1 (*m/z* = 355.10236)

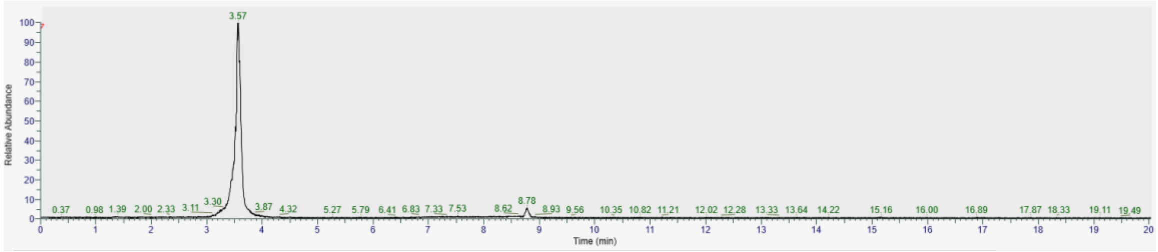

3.2 MS2

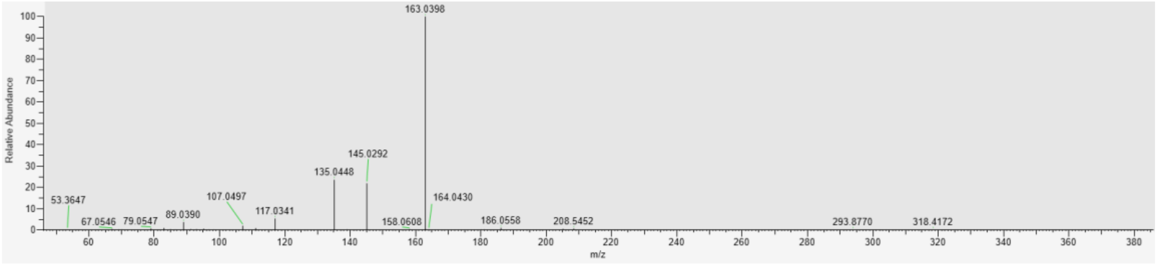

3.3 Reference MS2 spectrum obtained by mzCloud mass spectral database (10 Oct 2023)

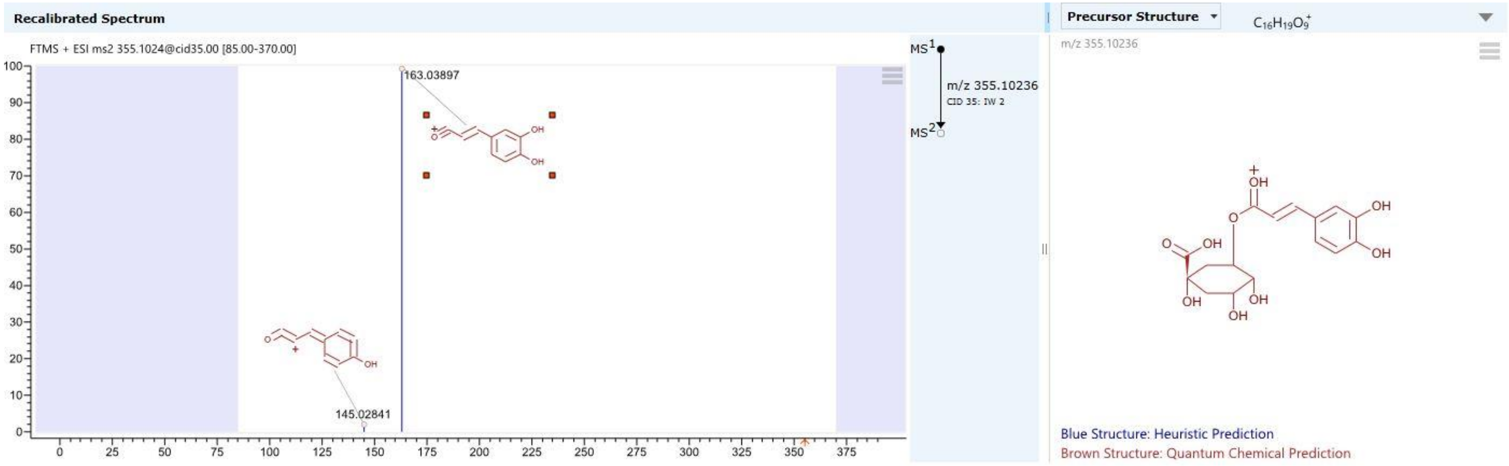

Supplement: Supplementary file 1 [file foods-12-03893-s001.zip › 13oct_Supplemetary data S1.pdf]
